# *In situ* visualization of m^6^A sites in cellular mRNAs

**DOI:** 10.1093/nar/gkad787

**Published:** 2023-10-09

**Authors:** Charles J Sheehan, Bahjat Fadi Marayati, Janvi Bhatia, Kate D Meyer

**Affiliations:** Department of Biochemistry, Duke University School of Medicine, Durham, NC, USA; Department of Biochemistry, Duke University School of Medicine, Durham, NC, USA; Trinity College of Arts and Sciences, Duke University, Durham, NC, USA; Department of Biochemistry, Duke University School of Medicine, Durham, NC, USA; Department of Neurobiology, Duke University School of Medicine, Durham, NC, USA

## Abstract

*N* ^6^-methyladenosine (m^6^A) is an abundant RNA modification which plays critical roles in RNA function and cellular physiology. However, our understanding of how m^6^A is spatially regulated remains limited due to a lack of methods for visualizing methylated transcripts of interest in cells. Here, we develop DART-FISH, a method for *in situ* visualization of specific m^6^A sites in target RNAs which enables simultaneous detection of both m^6^A-modified and unmodified transcript copies. We demonstrate the ability of DART-FISH to visualize m^6^A in a variety of mRNAs across diverse cell types and to provide information on the location and stoichiometry of m^6^A sites at single-cell resolution. Finally, we use DART-FISH to reveal that m^6^A is not sufficient for mRNA localization to stress granules during oxidative stress. This technique provides a powerful tool for examining m^6^A-modified transcript dynamics and investigating methylated RNA localization in individual cells.

## Graphical Abstract

**Graphical Abstract:**
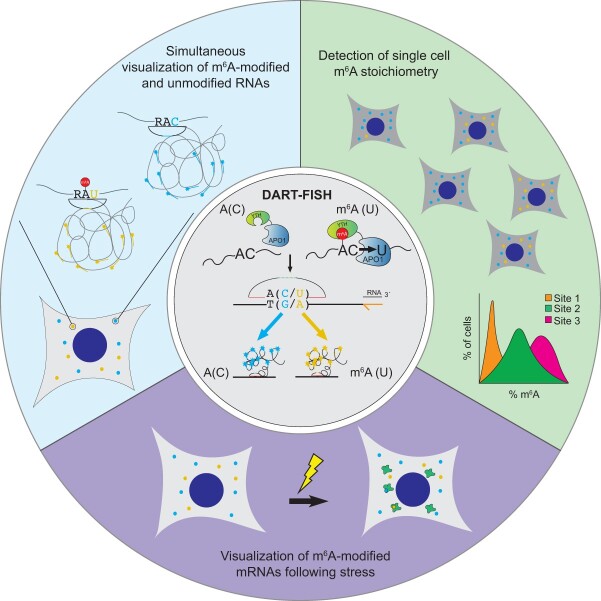

## Introduction

RNA modifications are critical regulators of gene expression. *N*^6^-Methyladenosine (m^6^A) is the most abundant internal mRNA modification and plays diverse roles in RNA function and processing, including the control of splicing, stability, and translation (1,2). As a result, m^6^A is important for a variety of physiological processes, such as stem cell differentiation, gametogenesis, immune responses, and learning and memory (1,2). In addition, improper regulation of m^6^A contributes to several diseases, including a variety of human cancers (1,2). Thus, identifying methylated RNAs in cells and understanding how they are regulated is important for determining how m^6^A influences gene expression under both physiological and pathogenic states.

To date, most studies examining cellular m^6^A have relied on transcriptome-wide m^6^A profiling or other methods that require cell lysis. This is a major barrier for our understanding of m^6^A since such approaches provide no information on m^6^A in its native context within the cell. Understanding where methylated and non-methylated mRNAs reside within the cell is particularly important since several studies have suggested that m^6^A-modified mRNAs can have a distinct subcellular localization compared to their unmodified counterparts (3–8). Additionally, the subcellular context in which an mRNA resides can have important implications for the function of that mRNA as well as the quantity and localization of protein it produces (9). Thus, tools for visualizing m^6^A in cells are greatly needed.

Although m^6^A antibodies can potentially be utilized for immunofluorescence-based detection of methylated RNAs, their cross-reactivity with the m^6^A_m_ modification precludes their use for visualizing m^6^A specifically. Additionally, strategies that detect m^6^A generically cannot provide information on single transcripts or distinguish m^6^A in rRNA from m^6^A in mRNAs or other RNA species. To overcome this, m^6^AISH-PLA was recently developed, which couples antibody-based m^6^A recognition with *in situ* hybridization and proximity ligation (10). However, this method only detects methylated sites and not their non-methylated counterparts, which limits its utility for analyzing differential localization of modified and unmodified transcripts or measuring m^6^A stoichiometry. Other approaches that have been developed include the use of nanoneedles conjugated with m^6^A antibodies to extract methylated RNAs from living cells while maintaining information on subcellular localization (11), or the use of oligo(dT)-coated coverslips followed by m^6^A antibody treatment and TIRF microscopy to visualize individual mRNAs and determine their methylation status (12). However, these methods require specialized materials, and they do not enable simultaneous visualization of methylated and unmethylated transcripts at specific m^6^A sites of interest. Thus, the development of simple methods for simultaneously visualizing m^6^A-modified and unmodified transcripts at base resolution remains a major unmet need in epitranscriptomics research.

Traditional methods for visualizing RNAs within cells involve the hybridization and subsequent detection of oligonucleotides complementary to the target sequence (13). However, these approaches are generally insensitive to single-nucleotide differences and RNA modifications. More recently, strategies that rely on differences in probe hybridization kinetics or ligation-based discrimination have been developed for detection of single-nucleotide variants (SNVs) (14,15). These methods have been used to visualize adenosine-to-inosine (A-to-I) editing, but they are not effective for detecting modifications like m^6^A that do not cause a change in Watson-Crick base pairing (16,17).

Previously, our group developed DART-seq (deamination adjacent to RNA modification targets), an antibody-free m^6^A detection method (18,19). DART-seq utilizes a fusion protein, APO1-YTH, which consists of the C-to-U deaminase APOBEC1 tethered to the m^6^A-binding YTH domain. When APO1-YTH is expressed in cells, it directs C-to-U deamination of cytidine residues which follow nearly all m^6^A sites. Although we initially developed DART-seq as a tool for global m^6^A mapping, we reasoned that we could leverage the power of DART-mediated editing by combining it with mutation-selective fluorescence *in situ* hybridization (FISH) to develop a method for visualizing m^6^A residues within cellular mRNAs.

Here, we present DART-FISH, a method that couples m^6^A-adjacent cytidine deamination with padlock probe (PLP) hybridization and rolling circle amplification to enable *in situ* visualization of m^6^A-modified transcripts in cells. We demonstrate the ability of DART-FISH to detect m^6^A-containing transcripts in a METTL3-dependent manner and show the utility of DART-FISH for investigating site-specific m^6^A modification across cell types and in distinct transcript isoforms. Additionally, we use DART-FISH to examine the subcellular localization of unmodified and m^6^A-modified mRNAs and determine that m^6^A methylation is not sufficient for transcript localization to stress granules following oxidative stress.

## Materials and methods

### Biological resources

#### Cell culture and treatments

All cell lines were maintained at 37°C with 5% CO_2_ using Dulbecco's modified Eagle's medium supplemented with 10% fetal bovine serum, 10 units/ml Penicillin, and 10 mg/ml Streptomycin. Cell lines were passaged at 80–90% confluence using TrypLE Express (Sigma). For imaging experiments, cells were plated onto poly-d-lysine (Gibco) coated coverslips at lower density. For HEK293T single-cell DART-FISH imaging experiments, cells were split into 10 cm plates, grown to 80% confluence and transgene expression induced with 1 μg/ml doxycycline overnight. Cells were passaged as described above. Cells were triturated to a single-cell suspension, filtered through a 40 μm filter (Corning), and plated onto poly-d-lysine (Gibco) coated #1.5 coverslips (Electron Microscopy Sciences/VWR). Cells were incubated for 8–12 h to recover prior to collection as described. For STM2457 treatment, cells were treated with 30 μM STM2457 (WuXi AppTech) or DMSO for 72 h prior to the addition of 1 μg/ml of doxycycline to the media and then incubated for an additional 24 h prior to collection. For siRNA treatment, HeLa cells were plated for 24 h prior to transfection of siRNAs targeting METTL3 and METTL14 at a 1: 2 ratio (Qiagen, 1027417:SI04317096, 1027417:SI00459942). siRNAs were transfected using Lipofectamine RNAiMax (ThermoFisher) following the manufacturer's protocol. Following 48 hrs of siRNA treatment, 1 μg/ml doxycycline was added to the media for 24 h prior to collection. Arsenite stress was induced following 24 h of doxycycline (1 μg/ml) induction by the addition of 1 ml of media containing water or 1 mM sodium arsenite (VWR, 0.1M) to cells in 1 ml of culture media for a final concentration of 500 μM. Cells were incubated for 1 h at 37°C with 5% CO_2_ prior to collection.

#### Stable cell line generation

Inducible APOBEC1-YTH or APOBEC1-YTH^mut^ stable cell lines were generated by infecting HeLa, NIH3T3, Neuro2a, and mHippoE-2 cells with APOBEC1-YTH-T2A-EGFP or APOBEC1-YTH^mut^-T2A-EGFP lentivirus (Addgene plasmid #178949 and #178950). U-2 OS G3BP1-GFP (a gift from Dr Paul Taylor) cells were infected with TLCV2 APOBEC1-YTH lentivirus with no EGFP tag. Cells were infected with 1:500 dilution of the respective lentivirus and following 48 h of infection the media was changed to media containing 2 μg/ml puromycin for 72 h. HeLa cells were clonally selected by serial dilution into a 96-well plate with 2 μg/ml puromycin-containing media. Following selection, stable cell pools were moved to standard media to recover before further analysis. Cell lines were validated by RT-PCR/Sanger sequencing and immunoblotting. To produce lentivirus, a 15 cm plate of HEK293T cells at 80% confluency was transfected with 26.75 μg of the respective plasmid, 20 μg of psPAX2 (Gift of Didier Trono, Addgene plasmid #12260), and 6.25 μg of pMD2.G (Gift of Didier Trono, Addgene plasmid #12259) using 3 mg jet-PEI (Polyplus). Seventy-two hours after transfection, the medium was collected and centrifuged at 5000 × g for 10 min. The supernatant was then filtered through a 0.45 mm filter into an ultracentrifuge tube (Beckman-Coulter). 4 ml of sterile 20% sucrose was added below the medium using a 5 ml serological pipette. The mixture was centrifuged at 19 700 rpm for 2 h at 4°C in a swinging bucket rotor (Beckman-Coulter, SW28). The supernatant was then removed, 100 μl of PBS was added, and the virus was resuspended overnight at 4°C with rocking. The resuspended virus was stored at −80°C.

### Constructs and cloning

Gibson assembly was used to generate all described constructs using NEBuilder HiFi DNA Assembly mix following the manufacturer's protocol (New England Biolabs). For *ACTB* reporter RNAs, the full-length *ACTB* transcript was amplified from HEK293T cDNA and mNeonGreen inserted at the 5′end of the coding sequence. Fragments were assembled into the pCMV backbone. To induce the U1223 point mutation, a fragment was amplified using a primer carrying the mutation and assembled into the pCMV backbone. To generate TLCV2-APO1-YTH, the APO1-YTH transgene was amplified from pCMV-APOBEC1-YTH (Addgene #131636) and inserted into the TLCV2 backbone (gift from Adam Karpf, Addgene #87360) replacing the CAS9-FLAG sequence.

### Reagents

#### Antibodies

The following antibodies and concentrations were used: rabbit anti-HA (Cell Signaling; 3724S; 1:1000), mouse anti-β-actin (Genscript; A00702; 1:1000), rabbit anti-GAPDH (Proteintech, 10494-1-AP, 1:1000), mouse anti-G3BP1 (Abcam, ab56574, 1:1000), rabbit anti-METTL3 (Abcam, ab195352, 1:1000), rabbit anti-METTL14 (Atlas, HPA038002, 1:1000), LI-COR IRDye® 680RD Goat anti-Mouse IgG Secondary Antibody (LiCor, 926–68070, 1:15000), LI-COR IRDye® 800RD Goat anti-Rabbit IgG Secondary Antibody (LiCor, 926-32211, 1:15000), AlexaFluor488-conjugated goat anti-rabbit (Thermo-Fisher; A-21206; 1:500), AlexaFluor568-conjugated goat anti-rabbit (Thermo-Fisher; A-11036; 1:500), AlexaFluoro488-conjugaed goat anti-mouse (Thermo-Fisher, A-11029, 1:500).

#### Immunoblotting

Cells were quickly rinsed with 1× PBS, incubated in TrypLE Express (Sigma,12604039) for 5 min at 37°C, suspended in cold 1× PBS, pelleted by centrifugation and pellets frozen at –80°C. Cell pellets were resuspended in lysis buffer (50 mM Tris–HCl, pH7.4; NaCl 100 mM; 1% NP-40 substitute (v/v); sodium dodecyl sulfate 0.1% (v/v); sodium deoxycholate 0.5% (v/v); cOmplete proteinase inhibitor cocktail (Sigma-Aldrich), 1 kU/ul benzonase (Sigma-Aldrich) and incubated on ice for 30 min. Lysates were then centrifuged at 21 630 rcf for 30 min at 4°C. The supernatant was transferred to a new tube. Samples for SDS-PAGE were then prepared at a final concentration of 1 mg/ml total protein in 1x NuPAGE LDS Sample Buffer (Invitrogen) and 0.1 M DTT (VWR). Samples were run on 4–12% SDS-PAGE gels (Invitrogen) and transferred for 90 min at 100 V in transfer buffer (25 mM Tris Base, 192 mM Glycine, 20% methanol (v/v)) to a nitrocellulose membrane (GE Amersham). After transferring, the membrane was blocked in PBST (PBS with 0.1% Tween 20 (Sigma-Aldrich)) with 5% milk (w/v) (Quality Biological) for >30 min at room temperature. Primary antibodies were incubated with the blots overnight at 4° in 5% milk. The membrane was washed 3 times with PBST before the secondary antibody was added for 45 min at room temperature in PBST. The membrane was then washed 3 times with PBST for 5 min. Blots were imaged on a Chemidoc MP (BioRad). Western blots were quantified in FIJI (2.9.0) (20). Pixel intensities were measured for each band using a set region of interest of defined area. Pixel intensities were inverted and normalized as a ratio to the loading control.

#### RNA extraction and cDNA synthesis

Cells were quickly rinsed with 1× PBS, incubated in TrypLE Express (Sigma,12604039) for 5 min at 37°C, suspended in cold 1× PBS, pelleted by centrifugation and pellets frozen at –80°C. RNA was extracted from cell pellets using the RNAeasy Mini Kit (Qiagen) following the manufacturer's protocol and eluted in 30 μl of RNAse-free water. RNA was quantified by nanodrop and cDNA was synthesized using 100–500 ng of total RNA with iScript SuperMix (BioRad).

#### RT-PCR and Sanger sequencing

cDNA was diluted 1:5 in water, and 1ul of the dilution was used for PCR amplification with CloneAmp™ HiFi PCR Premix (Takara). PCR primers are listed in Supplementary Table S1. PCR products were run on an agarose gel and purified using the QIAquick Gel Extraction Kit (Qiagen). PCR amplicons were directly sequenced using Sanger sequencing (Azenta). C-to-U conversion was measured using EditR software (21).

#### 3′RACE and sequencing of *Actb* isoforms

cDNA was synthesized using 150ng of total RNA with SuperScript III reverse transcriptase (ThermoFisher) following the manufacturer's protocol. RT was primed using 50 pmol of a 17-mer oligo(dT) primer with an adapter sequence on the 5′end. *Actb* transcripts were amplified from 1 μl of a 1/5 dilution of the 3′RACE cDNA using a gene-specific forward primer and adapter-targeting reverse primer (see Supplementary Table S1). 1 μl of a 1/5 dilution of this PCR reaction was used as input into a nested PCR to amplify each isoform. Amplicons were purified by gel extraction and sent for sequencing. The long *Actb* isoform was sequenced using Oxford Nanopore sequencing (Plasmidsaurus). Due to size limitations, the short isoform was sequenced using Sanger sequencing (Azenta).

#### RT-qPCR-based relative quantification of m^6^A

RNA was isolated from APO1-YTH HEK293T and HeLa stable cells without doxycycline induction using Trizol (Invitrogen) and then treated with DNAse I (New England Biolabs) for 1 h at 37°C. RNA was purified by alcohol precipitation. Two reverse transcription reactions were set up for each sample using BST polymerase (NEB); one with the m^6^A adjacent primer (+) and one using a non-adjacent primer (−). Reverse transcription reactions contained 1× ThermoPol Buffer (New England Biolabs), 50 μM dNTPs, 500 nM primer, 150 ng of total RNA and 10 U of BST polymerase. Reactions were incubated in a thermocycler using the following protocol: 2 min 25°C, 15 min 50°C and 3 min 85°C. Additionally, two reverse transcription reactions were set up using Superscript III (Invitrogen): one with the m^6^A adjacent primer and one with the non-adjacent primer and using150 ng of total RNA and 500 nM primer. Quantitative PCR was performed using complementary DNA with indicated primers (see Supplementary Table S1) and iQ SYBR Green Super Mix (BioRad) in a CFX Connect Real-Time PCR system (Bio-Rad) via Bio-Rad CFX Maestro 1.1. (4.1.2433.1219). Relative methylation was calculated with the obtained Ct values as previously described (22) with the following formula:


\begin{equation*}{\mathrm{Rel}}{\mathrm{. }}{{\mathrm{m}}}^6{\mathrm{A}} = {2}^{ - \left( {{\mathrm{Ct Bst}}\left( - \right){\mathrm{ }}--{\mathrm{ Ct SSIII}}\left( - \right){\mathrm{ }}/{\mathrm{ Ct Bst}}\left( + \right){\mathrm{ }}--{\mathrm{ Ct SSIII}}\left( + \right)} \right)}.\end{equation*}

#### DART-FISH

Cells grown on coverslips were washed in cold 1× PBS and fixed in 4% paraformaldehyde in PBS (Fisher Scientific) for 10 min at room temperature. Coverslips were then washed once in 2× SSC followed by permeabilization and storage in 70% ethanol for >1 h. Coverslips were then removed from 70% ethanol, washed once in 0.05% PBST, and incubated for 10 min at room temperature in 0.1M hydrochloric acid (HCl) to make RNA more readily available for cDNA synthesis. Coverslips were then wash twice in 1× PBS for 3 min each followed by preincubation in 1× RevertAid H-minus Reverse Transcriptase reaction buffer (ThermoFisher). Coverslips were placed cell-side down onto a layer of parafilm each in 50 μl reverse transcription mix (1× RevertAid H-minus RT reaction buffer, 0.5 mM dNTPs (New England Biolabs), 0.2 μg μl^−1^ BSA (Invitrogen), 1 U μl^−1^ RiboLock RNAse Inhibitor (ThermoFisher), 1 μM cDNA LNA primer, 5 U μl^−1^ RevertAid H-minus Reverse Transcriptase (ThermoFisher)). Reverse transcription was carried out for 1 h at 37°C in a humidified chamber wet with 2× SSC.

Following reverse transcription, coverslips were washed twice in 0.05% PBST and fixed in 4% paraformaldehyde in PBS (Fisher Scientific) at room temperature for 10 min. Coverslips were again washed twice in 0.05% PBST then preincubated in 1× Ampligase Reaction Buffer (Biosearch Technologies). Coverslips were placed cell-side down on parafilm in a 2X SSC humidified chamber each with 50ul of hybridization/ligation mix (1× Ampligase Reaction Buffer, 20% Formamide (VWR), 5 mM KCl, 0.4U μl^−1^ RNAse H (Takara), 1U μl^−1^ RiboLock RNAse Inhibitor (ThermoFisher), 0.1U μl^−1^ Ampligase Ligase (Biosearch Technologies)) containing 100 nM of each padlock probe. Hybridization/ligation was carried out by incubating samples for 30 min at 37°C followed by 45 min at 45°C.

Following hybridization/ligation, coverslips were washed twice in 0.05% PBST then preincubated in Phi29 DNA Polymerase Buffer (ThermoFisher). Rolling circle amplification (RCA) was conducted for 100 min at 37°C with coverslips cell-side down on parafilm in 50 μl of RCA mix (1X Phi29 DNA Polymerase Buffer, 5% glycerol, 0.5 mM dNTPs (New England Biolabs), 0.2 μg BSA (Invitrogen), 1 U μl^−1^ RiboLock RNAse Inhibitor (ThermoFisher), 1 U μl^−1^ Phi29 DNA Polymerase (ThermoFisher). Following RCA, coverslips were washed once in 0.05% PBST. Rolling circle products (RCPs) were visualized by incubating coverslips cell-side down on parafilm in a humidified chamber for 30min at 37°C in 50 μl of detection mix (2× SSC, 20% formamide, 1μg/ml DAPI) containing 100nM of each detection probe. Coverslips were washed once in 0.05% PBST followed by 2× PBS washes. Coverslips were mounted onto slides using Vectashield Vibrance (Vector Laboratories). The DART-FISH protocol can be completed in a single day: from the start of reverse transcription through coverslip mounting, the protocol takes approximately 5–6 h to complete.

For experiments coupling DART-FISH with stress granule visualization, samples were co-stained for G3BP1 using immunofluorescence due to decreased GFP fluorescence following sample processing during DART-FISH. Following rolling circle amplification, coverslips were washed twice in PBS at room temperature. Coverslips were incubated with primary antibody in 0.05% PBST overnight at 4°C. Coverslips were washed 3X in PBS then incubated with the secondary antibody in 0.05% PBST for 45 min at room temperature. Samples were washed 3× with PBS then fixed in 4% PFA for 10 min at room temperature. Following fixation, RCPs were visualized as described above.

#### Immunofluorescence

Cells grown on coverslips were washed in cold 1× PBS and fixed in 4% paraformaldehyde in PBS (Fisher Scientific) for 10 min at room temperature. Coverslips were then washed once in 2× SSC followed by permeabilization and storage in 70% ethanol for >1 h. Cells were removed from 70% ethanol and washed three times in 1× PBS. Coverslips were blocked in 1% bovine serum albumin (BSA) (VWR) for >30 min. Samples were incubated with primary antibody in 1% BSA overnight at 4°C, washed three times, then incubated with the secondary antibody and 1 μg/ml DAPI in 1% BSA at room temperature for 45 min. Coverslips were washed three times in 1× PBS prior to mounting with Vectashield Vibrance (Vector Laboratories). Images were acquired using uniform exposure times and processing settings across conditions. Representative images have uniform adjustments across all displayed channels except for DAPI signal which may be differentially adjusted to account for variable intensity across different cell types.

#### smFISH

Oligonucleotides complementary to human *ACTB* were designed using Stellaris Probe Designer and ordered from Biosearch Technologies conjugated to Cal 610 with dual HPLC purification. Sequences are provided in Supplementary Table S1. Probes were resuspended at a final concentration of 12.5 μM. To perform smFISH, cells grown on coverslips were washed in cold 1× PBS and fixed in 4% paraformaldehyde in PBS (Fisher Scientific) for 10 min at room temperature. Coverslips were then washed once in 2× SSC followed by permeabilization and storage in 70% ethanol for > 1hr. Coverslips were then removed from 70% ethanol storage and equilibrated at room temperature in FISH-WASH buffer (2× SSC with 10% formamide). For each sample, 50ul of hybridization buffer was prepared containing: 1 μl smFISH probe set, 2× SSC, 10% formamide, and 10% dextran sulfate. Coverslips were incubated in hybridization buffer overnight at 37°C. Coverslips were then washed three times in FISH-WASH: once at room temperature for 3 min, once for 30 min at 37°C, and once for 30 min at 37°C containing 1μg/ml DAPI. Following washes, coverslips were rinsed in 2× SSC then mounted using Vectashield Vibrance (Vector Laboratories).

#### Image acquisition

Images were acquired using an inverted wide-field Leica DMi8 microscope equipped with a 63×/1.4 HC PL APO objective, Leica DFC9000 4.2 MP monochrome sCMOS camera, Lumencor SOLA SM light engine, and the following filter cubes: DAPI (EX350/50; DC400; EM460/50), GFP (EX470/40; DC495; EM525/50), RHOD (EX546/10; DC560; EM585/40) and Y5 (EX620/60; DC660; EM700/75). The Leica LasX software was used for image acquisition and initial processing. For each field of view, a 3D image stack starting below and ending above detectable DAPI signal was captured with a z-step size of 210 nm at 12-bit image depth. The resulting image voxel sizes were (*x* × *y* × *z*) 0.105 nm × 0.105 nm × 0.210 nm. Exposure times were set and maintained for all samples within an experiment.

#### Image quantification and analysis

Images were 3D-deconvoluted in the Leica LasX software using the AutoQuant algorithm to remove background signal and resize to 16bit-depth using a refractive index of 1.47 to match that of Vectashield Vibrance. z-stacks were trimmed in FIJI (2.9.0) (20) to remove the first and last 10 z-planes, then transformed to maximal intensity projections. Individual cells were identified and masked using CellProfiler (4.0.5) (23). DART-FISH RCPs and smFISH signal were both quantified using FISH-quant (24) run through Matlab (9.8). 3D z-stacks were quantified by channel for individual mRNA spots and non-specific signal was filtered out using signal amplitude and width in *x–y* and *x*–*z* dimensions. Individual channel settings were set and then all images for a single experiment were analyzed in an automated fashion. The number of mRNAs per cell from each channel was exported and visualized in RStudio running R (4.1.0) and GraphPad Prism (9.0.5).

Only cells with 10 or greater total RCPs were kept for downstream analysis. Representative images in figures are maximal intensity projections unless otherwise indicated. Image contrast is adjusted to reduce background signal.

For DART-FISH optimization experiments in HEK293T cells, images were quantified as a whole field of view. Images were acquired and processed as above, then maximal intensity projections were used as input into CellProfiler. Each channel was quantified as a single field of view. Nuclei were identified using DAPI staining and called as primary objects. RNA puncta were called as secondary objects based on intensity and size. The total number of mRNA molecules per channel and number of nuclei was exported and processed in Microsoft Excel (16.68). The average number of RCPs per cell was calculated by determining the total number of RCPs divided by the number of cells (as determined by DAPI stain) in the field of view.

#### Lattice structured illumination (SIM) microscopy

Lattice-SIM images were acquired on a Zeiss Elyra 7 AxioObserver microscope equipped with an Plan-Apochromat 63×/1.4 Oil DIC M27 objective and two pco.edge sCMOS (version 4.2 CL HS) cameras which were aligned at the start of the experiment daily. The system contained 405 nm and 642 nm diode, and 488 nm and 561 nm OPSL lasers. For each focal plane, 13 phase images were acquired. Exposure time and laser power were balanced for each fluorescence channel individually to minimize bleaching and exposure time set to 50ms for all channels. The lasers and filters to acquire each fluorophore are as follows: For 4-channel DART-FISH: DAPI: 405 nm at 5% with BP 420–480 + BP 495–550; 488: 488 nm at 1% with BP 420–480 + BP 495–550; Cy3: 561 nm at 4% with BP 570–620 + LP 655; Cy5: 642 nm at 3% with BP 570–620 + LP655. For 3-channel smFISH: DAPI: 405nm at 3% with BP 420–480 + BP 495–550; 488: 488 nm at 2% with BP 420–480 + BP 495–550; TexasRed: 561nm at 8% with BP 570–620 + LP 655 and 75 ms exposure time. The DAPI channel was separated from z-stack prior to SIM processing. SIM processing and reconstruction was performed with the SIM processing tool of the ZEN 3.0 SR (black) software with the following settings. 3D processing, AutoSharpness, baseline, scale to raw image, and channel alignment set when initially calibrating the microscope. DAPI Images were processed under identical conditions except for channel alignment.

SIM-processed 3D z-stacks were used as input into Imaris (Version 9.9.1) for quantification. Channels were masked to contain only signal from a single cell by generating a surface using a hand drawn ROI of the cell of interest. Masked channels were then used to generate stress granule (G3BP1) surfaces using a surface detail of 0.1 μm and absolute intensity to discriminate individual granules. Stress granules smaller than 10 voxels were removed from analysis. RCPs or smFISH signal were designated using the respective channel with background subtraction to identify mRNAs. Final mRNAs were filtered manually using quality score. Statistics for each individual mRNA molecule were exported and visualized in RStudio running R (4.1.0) and GraphPad Prism (9.0.5). Molecules for which the shortest distance to the nearest stress granule was less than or equal to 0 μm were called as colocalized.

#### scDART-seq Analysis

The raw data from scDART-seq in HEK293T cells was processed as previously described (19). To identify C-to-U editing sites from matrix files, find_RNA_edit_sites was run using –KnownSites with a bed file containing the hg38 genome coordinates of m^6^A sites targeted by DART-FISH (see Supplementary Table S2). Edit sites were called in all cells looking for C-to-T mutations against the hg38 genome build with a minimum editing of 0, maximum editing of 100, minimum coverage of 1, and minimum edit sites of 0. Editing events in all cells were concatenated into a single table and exported for visualization in RStudio running R (4.1.0) and GraphPad Prism (9.0.5).

#### Statistical analyses

For imaging-based experiments, *n* represents the number of individual cells tested across a minimum of two independent biological replicates with at least three independent fields of view sampled. As noted above, the DART-FISH optimization experiments in HEK293T cells were quantified as entire fields of view with *n* representing each independent biological replicate with at least 3 independent technical replicates (i.e. images) quantified per sample. For all other analyses, *n* indicates the number of biological replicates tested.

When noted, *P*-values represent values calculated as follows: for single variable analysis with only two conditions, a two-tailed unpaired Student's *t*-test was applied. For single variable analysis with greater than two conditions (i.e. treatment across RCP types), a one-way ANOVA was applied. For two-variable analysis (i.e. treatment and cell type), a two-way ANOVA with Tukey post-hoc test was applied.

## Results

### DART-FISH enables visualization of m^6^A-modified RNAs in cells

**Figure 1.**
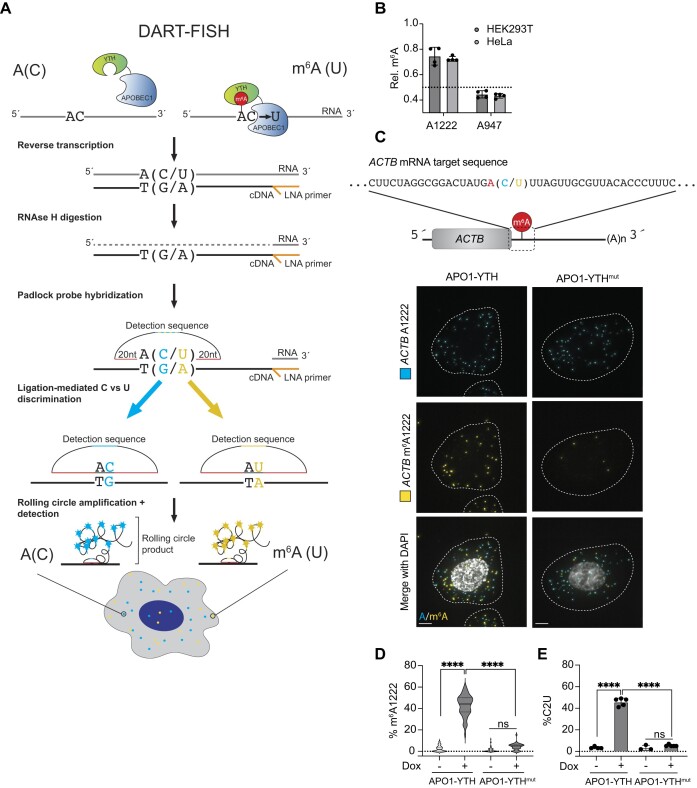
Overview of the DART-FISH method. (**A**) Schematic representation of DART-FISH. Exogenous expression of APO1-YTH directs C-to-U deamination of cytidine residues adjacent to m^6^A sites. Cells are collected, fixed, and undergo *in situ* reverse transcription using a targeted locked nucleic acid (LNA) primer. The RNA molecule is digested by RNAse H, and padlock probes complementary to the target sequence with either a C or U adjacent to the m^6^A site of interest are hybridized to the cDNA. The 5′ and 3′ ends of circularized padlock probes are then ligated together, which is only achieved with complete complementarity to the target sequence. Ligated padlock probes are amplified using rolling circle amplification and visualized via hybridization of fluorescent oligonucleotides complementary to the unique detection sequence within each padlock probe. (**B**) RT-qPCR-based m^6^A quantification at the *ACTB* A1222 site in HEK293T and HeLa cells. An unmethylated adenosine, *ACTB* A947, is included as a negative control site. Dotted line indicates the threshold value (0.5) for the presence of m^6^A. Values shown are mean ± s.d.; *n* = 4. (**C**) Top: Schematic representation of the *ACTB* mRNA with the 40nt padlock probe target sequence shown in the zoomed in region. The red adenosine residue indicates the m^6^A site at *ACTB* A1222. At position 1223 both the C (unmethylated) and U (methylated) versions of the transcript are shown. Bottom: Representative images of DART-FISH targeting *ACTB* m^6^A1222 in doxycycline-treated HeLa cells expressing APO1-YTH or APO1-YTH^mut^. Dotted lines indicate cell outlines. Scale bar = 5 μm. (**D**) Quantification of the proportion of *ACTB* transcripts with m^6^A1222 detected in HeLa cells with or without doxycycline induction of APO1-YTH or APO1-YTH^mut^. The percentage of m^6^A1222 transcripts in individual cells is plotted. Solid line represents median with quartiles shown by dotted lines. APO1-YTH -dox: *n* = 67; APO1-YTH + dox: *n* = 52; APO1-YTH^mut^ -dox: *n* = 53; APO1-YTH^mut^ + dox: *n* = 36. ****P < 0.0001, ns = not statistically significant. (**E**) %C2U deamination assessed by RT-PCR and Sanger sequencing in the indicated HeLa stable cell lines with or without doxycycline induction (APO1-YTH - dox: *n* = 4; APO1-YTH + dox: *n* = 5; APO1-YTH^mut^ -dox: *n* = 3; APO1-YTH^mut^ + dox: *n* = 5). Values shown are mean ± s.d. ****P < 0.0001; ns = not statistically significant.

To develop DART-FISH, we combined APO1-YTH expression with PLP hybridization and rolling circle amplification to visualize the presence or absence of m^6^A at an individual site of interest (Figure 1A). First, APO1-YTH marks m^6^A sites in cellular mRNAs by binding to m^6^A and directing C-to-U editing at adjacent cytidine residues. Cells are then fixed, followed by reverse transcription and RNase H digestion. Detection of C or U transcript variants is achieved by the hybridization of SNV-selective PLPs to the target cDNA for an RNA of interest. The circularized 5′ and 3′ ends of hybridized PLPs are then ligated together, which requires perfect complementarity to the target nucleotides and therefore enables specific detection of SNVs (Figure 1A) (15). Following PLP ligation, rolling circle amplification of the ligated padlock probe generates a repetitive detection element which is then visualized by the hybridization of fluorescent oligonucleotides (Figure 1A). Thus, by designing probes that target the C and U variants adjacent to an m^6^A site of interest, we can detect both variants simultaneously with distinct fluorophores. Since APO1-YTH selectively installs C-to-U mutations adjacent to methylated, but not unmethylated, adenosines, this strategy enables simultaneous visualization of methylated (U) and unmethylated (C) transcripts in cells (Figure 1A).

To determine whether DART-FISH can selectively detect m^6^A-modified mRNA, we targeted an m^6^A site within the 3′UTR of human *ACTB* at position A1222 which has been identified in several m^6^A mapping datasets (18,19,25,26). We validated methylation at *ACTB* A1222 in both HEK293T and HeLa cell lines using RT-qPCR-based m^6^A quantification (22) (Figure 1B). When A1222 is methylated, expression of APO1-YTH produces the predicted C-to-U mutation at position 1223 (Supplementary Figure S1A). To visualize methylated and unmethylated *ACTB* transcripts, we designed PLPs targeting the U1223 and C1223 variants, respectively. Probe specificity for each variant was confirmed by overexpressing human *ACTB* reporter mRNAs containing either a C or U at position 1223 in mouse NIH3T3 cells. The variant-specific PLPs were highly selective for their targets despite the difference of only a single nucleotide, and the probes showed no background hybridization to mouse *Actb* (Supplementary Figure S1B, C). We then tested various hybridization and rolling circle amplification conditions to develop an optimized DART-FISH protocol (Supplementary Figure S1D–G).

To determine whether DART-FISH can be used to visualize methylated and non-methylated forms of endogenous *ACTB*, we generated stable HeLa cell lines with doxycycline-inducible expression of APO1-YTH and confirmed doxycycline-dependent C-to-U editing adjacent to m^6^A1222 in *ACTB* (Supplementary Figure S1H, I). As a control, we also generated stable cell lines inducibly expressing APO1-YTH^mut^, which has a truncated YTH domain and exhibits reduced m^6^A binding (18,27). Editing of *ACTB* C1223 was greatly diminished in these cells, further confirming that APO1-YTH-mediated C-to-U editing at this position depends on recognition of m^6^A (Supplementary Figure S1I). We then used DART-FISH to target methylated (U1223) and unmethylated (C1223) *ACTB* in HeLa cells expressing either APO1-YTH or APO1-YTH^mut^. As expected, we detected the unmethylated *ACTB* transcript in both the APO1-YTH and APO1-YTH^mut^-expressing cell lines (Figure 1C, D). In contrast, the methylated transcript was readily detected in APO1-YTH-expressing cells but showed only negligible levels in APO1-YTH^mut^-expressing cells (Figure 1C, D, Supplementary Figure S1J). 43% of the *ACTB* transcripts detected in APO1-YTH-expressing cells contain U1223, which mirrors the level of C-to-U editing adjacent to m^6^A1222 as measured by RT-PCR and Sanger sequencing (Figure 1E). Thus, DART-FISH signal faithfully represents the APO1-YTH-mediated C-to-U editing rate observed adjacent to m^6^A1222, and it requires the m^6^A-binding ability of APO1-YTH.

**Figure 2.**
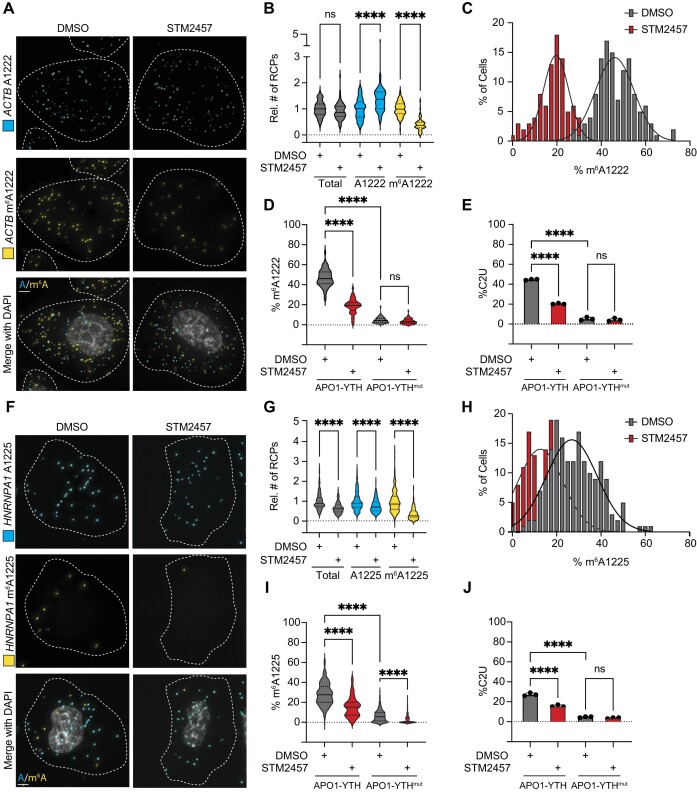
DART-FISH detection of m^6^A is METTL3-dependent. (**A**) Representative images of DART-FISH targeting the *ACTB* m^6^A1222 site in APO1-YTH-expressing HeLa cells treated with DMSO or STM2457. Dotted lines represent cell outlines. Scale bar = 5 μM. (**B**) Quantification of the number of methylated, nonmethylated, and total transcripts detected by DART-FISH targeting the *ACTB* m^6^A1222 site in HeLa cells treated with DMSO or STM2457. Values plotted are the number of RCPs in individual cells relative to the population average in DMSO-treated cells. Solid lines represent median with quartiles shown by dotted lines. ****P < 0.0001, ns = not statistically significant. (**C**) Histogram showing the proportion of *ACTB* m^6^A1222 transcripts detected in individual HeLa cells following DMSO or STM2457 treatment. Solid line represents Gaussian fit of the distribution. (**D**) Percentage of *ACTB* transcripts with m^6^A1222 as detected by DART-FISH in HeLa cells expressing APO1-YTH or APO1-YTH^mut^ following DMSO or STM2457 treatment. Solid lines represent median with quartiles shown by dotted lines. APO1-YTH DMSO: *n* = 123; APO1-YTH STM2457: *n* = 90; APO1-YTH^mut^ DMSO: *n* = 105; APO1-YTH^mut^ STM2457: *n* = 86 for panels B–D. ****P < 0.0001, ns = not statistically significant. (**E**) %C2U values adjacent to *ACTB* A1222 as determined by RT-PCR and Sanger sequencing of HeLa cells expressing APO1-YTH or APO1-YTH^mut^ following DMSO or STM2457 treatment. Values shown are mean ± s.d.; *n* = 3 for all conditions. ****P < 0.0001, ns = not statistically significant. (**F**) Representative images of DART-FISH targeting the *HNRNPA1* m^6^A1225 site in APO1-YTH-expressing HeLa cells treated with DMSO or STM2457. Dotted lines represent cell outlines; scale bar = 5 μm. (**G**) Quantification of the number of methylated, nonmethylated, and total transcripts detected by DART-FISH targeting the *HNRNPA1* m^6^A1225 site in HeLa cells treated with DMSO or STM2457. Values plotted are the number of RCPs in individual cells relative to the population average in DMSO-treated cells. Solid lines represent median with quartiles shown by dotted lines. ****P < 0.0001. (**H**) Histogram showing the proportion of *HNRNPA1* m^6^A1225 transcripts detected in individual HeLa cells following DMSO or STM2457 treatment. Solid line represents Gaussian fit of the distribution. (**I**) Percentage of *HNRNPA1* transcripts with m^6^A1225 as detected by DART-FISH in HeLa cells expressing APO1-YTH or APO1-YTH^mut^ following DMSO or STM2457 treatment. Solid lines represent median with quartiles shown by dotted lines. ****P < 0.0001. APO1-YTH DMSO: *n* = 177, APO1-YTH STM2457: *n* = 135, APO1-YTH^mut^ DMSO: *n* = 165, APO1-YTH^mut^ STM2457: *n* = 204 for panels G–I. (**J**) %C2U values adjacent to *HNRNPA1* A1225 as determined by RT-PCR and Sanger sequencing of HeLa cells expressing APO1-YTH or APO1-YTH^mut^ following DMSO or STM2457 treatment. Values shown are mean ± s.d.; *n* = 3 for all conditions. ****P < 0.0001, ns = not statistically significant.

To further confirm the m^6^A-dependence of DART-FISH, we treated APO1-YTH-expressing cells with STM2457, a small molecule inhibitor of METTL3 (28), and performed DART-FISH targeting *ACTB* A/m^6^A1222. We observed no significant change in the total number of *ACTB* transcripts following STM2457 treatment (Figure 2A, B). However, there was a nearly 60% reduction in the number of methylated transcripts in STM2457-treated cells and a corresponding increase in unmethylated transcripts (Figure 2A–D, Supplementary Figure S2A). The decrease in m^6^A1222 transcripts detected by DART-FISH once again matched the decrease in adjacent C-to-U deamination measured by RT-PCR and Sanger sequencing, further indicating the specificity of the DART-FISH signal (Figure 2E, Supplementary Figure S2B). In addition, we used siRNAs to deplete the m^6^A methyltransferase components METTL3 and METTL14 (Supplementary Figure S2C). Similar to STM2457 treatment, this led to a significant decrease in m^6^A1222 transcripts and a corresponding increase in A1222 transcripts, consistent with a reduction in m^6^A-modified transcripts and an increase in unmethylated transcripts (Supplementary Figure S2D–G). Altogether, these data demonstrate that DART-FISH can be used to visualize m^6^A residues in a METTL3-dependent manner and that it can detect changes in cellular m^6^A caused by METTL3 inhibition or METTL3/14 reduction.

Since the A1222 position in *ACTB* is a relatively frequently methylated site (19,26), we asked whether DART-FISH can be used to visualize m^6^A residues of lower abundance. We targeted an m^6^A site within the 3′UTR of *HNRNPA1* at position A1225, which is estimated to be approximately half as abundant as the m^6^A1222 site in *ACTB* (19). Indeed, DART-FISH revealed that an average of 28% of *HNRNPA1* transcripts are methylated at position A1225, compared to 43% for the A1222 position in *ACTB* (Figure 2F–I). Additionally, both STM2457 treatment and METTL3/14 depletion caused a significant decrease in DART-FISH signal at m^6^A1225 in *HNRNPA1*, once again indicating that DART-FISH is m^6^A-dependent (Figure 2F–J, Supplementary Figure S2H–L). Altogether, these data demonstrate that DART-FISH can be used to visualize m^6^A-modified sites of varying abundance in endogenous mRNAs at single-nucleotide and single-cell resolution.

### DART-FISH reveals heterogeneity in site-specific methylation across single cells

**Figure 3.**
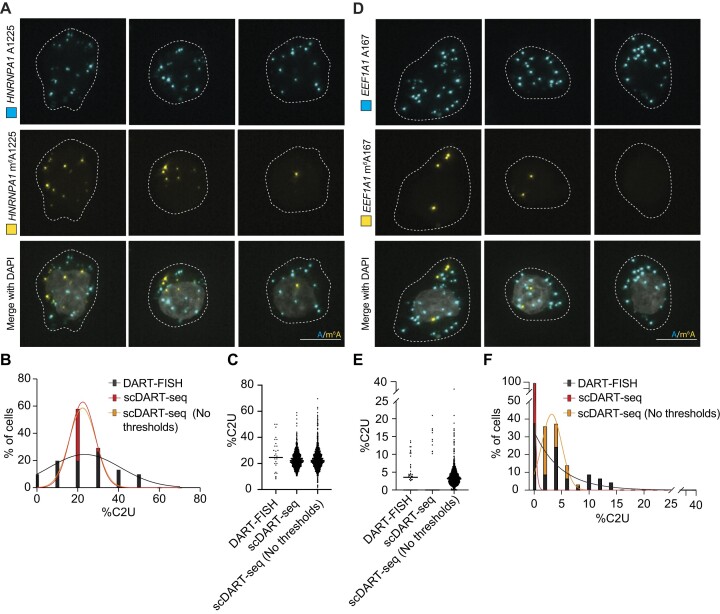
DART-FISH measures site-specific m^6^A methylation in single cells. (**A**) Representative images of DART-FISH targeting the *HNRNPA1* m^6^A1225 site in HEK293T cells. Each column depicts a single cell within the same field of view. Dotted lines represent cell outlines; scale bar = 10 μm. (**B**) Frequency distribution showing the %C2U values adjacent to *HNRNPA1* A1225 in individual HEK293T cells detected using DART-FISH, scDART-seq, or re-analysis of the scDART-seq dataset without stringency thresholds. Solid lines represent Gaussian fit of the distribution. DART-FISH: *n* = 30 cells; scDART-seq: *n* = 1068 cells; scDART-seq (no thresholds) *n* = 1254 cells. (**C**) Scatterplot of % C-to-U editing adjacent to A1225 in *HNRNPA1* as detected by DART-FISH, scDART-seq, or re-analysis of the scDART-seq dataset without stringency thresholds in individual cells. Solid line represents median. DART-FISH: *n* = 30 cells; scDART-seq: *n* = 1068 cells; scDART-seq (no thresholds): *n* = 1254 cells. (**D**) Representative images of DART-FISH targeting *EEF1A1* m^6^A167 in HEK293T cells. Each column depicts a single cell within the same field of view. Dotted lines represent cell outlines; scale bar = 10 μm. (**E**) Scatterplot of %C2U adjacent to *EEF1A1* A167 in individual HEK293T cells detected by DART-FISH, scDART-seq or re-analysis of the scDART-seq dataset without stringency thresholds. Solid line represents median. DART-FISH: *n* = 45; scDART-seq: *n* = 1068; scDART-seq (no thresholds): *n* = 1254. (**F**) Frequency distribution showing %C2U adjacent to *EEF1A1* A167 in individual HEK293T cells detected by DART-FISH, scDART-seq or re-analysis of the scDART-seq dataset without stringency thresholds. Solid line represents a Gaussian fit of the distribution. DART-FISH: *n* = 45; scDART-seq: *n* = 1068; scDART-seq (no thresholds): *n* = 1254.

Our previous work using scDART-seq to map m^6^A transcriptome-wide in single cells revealed that methylation frequency at individual sites in the same mRNA can be highly variable across cells (19). However, single-cell sequencing-based approaches suffer from dropout effects, and they do not enable direct visualization of methylated and unmethylated transcripts within cells. To determine whether DART-FISH can overcome these limitations, we used it to quantify methylated and unmethylated target mRNAs across individual cells. We chose two m^6^A sites of widely varying frequency: site A1225 in the 3′UTR of *HNRNPA1*, which we previously found to be methylated in over 98% of HEK293T cells, and site A167 in the coding sequence of *EEF1A1*, which we found to be methylated in only 1% of HEK293T cells (19). We performed DART-FISH in HEK293T cells targeting these two m^6^A sites and found that, consistent with our sequencing-based analysis, over 90% of cells contain m^6^A at *HNRNPA1* A1225 (Figure 3A–C, Supplementary Figure S3A). Additionally, the distribution of methylated and non-methylated transcripts across single cells was highly similar between DART-FISH and scDART-seq (Figure 3B). Interestingly, when we conducted DART-FISH targeting the m^6^A site at A167 in *EEF1A1*, we found that 44% of cells had methylation at this site, which is much higher than the 1% of cells that we originally reported using scDART-seq (Figure 3D–F, Supplementary Figure S3B). We reasoned that this discrepancy might be due to the thresholds used for calling m^6^A sites in scDART-seq data, which uses a cutoff of 10% C2U to call an m^6^A site (19). Indeed, when we quantified m^6^A167 using DART-FISH, we found that m^6^A is present at this site in fewer than 10% of transcripts per cell (Figure 3D, F). Additionally, reanalysis of our scDART-seq data without editing thresholds identified low-frequency methylation at *EEF1A1* A167 in a much greater proportion of cells (Figure 3E, F). We performed the same reanalysis of the scDART-seq dataset for site m^6^A1225 in *HNRNPA1*, which exhibits much higher levels of methylation across cells (Figure 3A–C). As expected, removing the site calling thresholds did not substantially change the proportion of cells identified as having *HNRNPA1* m^6^A1225 methylation, since most cells already exhibit %C2U values greater than 10% (Figure 3B, C). Altogether, these data demonstrate that DART-FISH can be used to visualize m^6^A methylation heterogeneity at single sites across individual cells of a population, including rare methylation events that can be missed by sequencing-based approaches.

### DART-FISH detects m^6^A in distinct transcript isoforms

**Figure 4.**
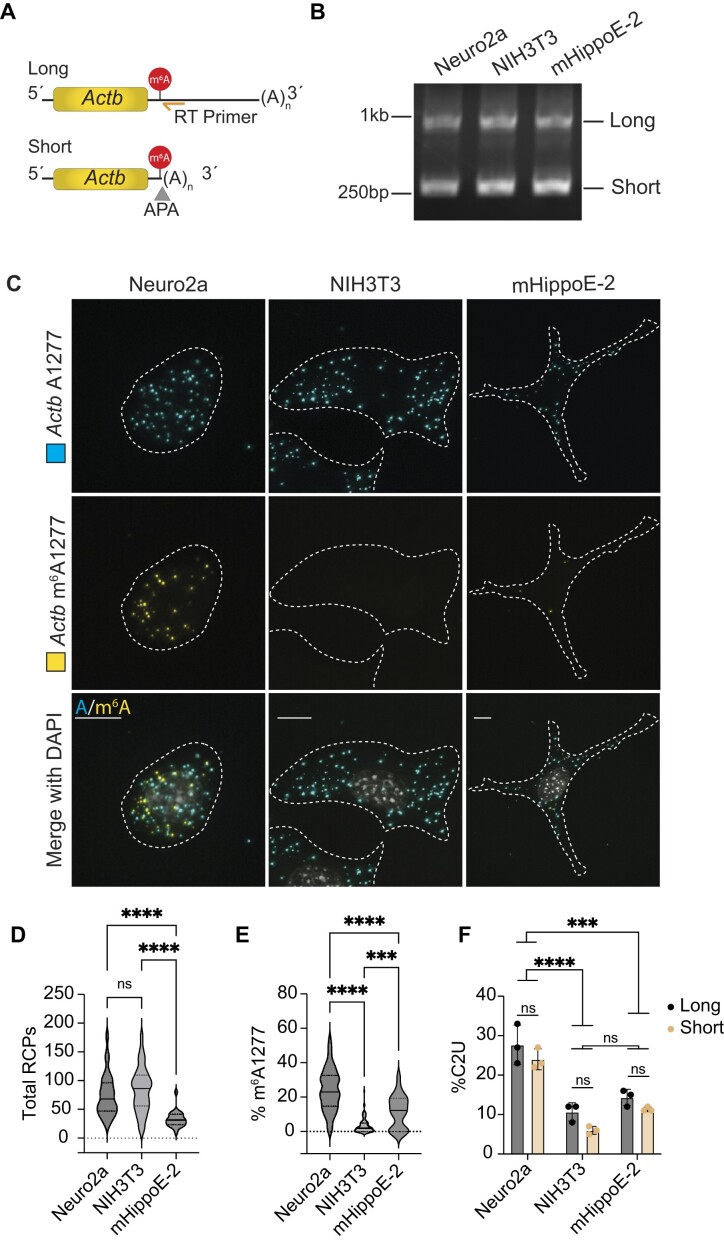
DART-FISH can detect m^6^A in distinct transcript isoforms. (**A**) Schematic showing the short and long *Actb* isoforms. The short isoform is generated by use of an upstream APA site (gray triangle) which is 19nt downstream from A1277. DART-FISH targets the long isoform specifically by using a reverse transcription primer (orange) which hybridizes downstream of the APA site. (**B**) Representative 3′RACE and PCR using a gene*-*specific forward primer to amplify *Actb* isoforms in APO1-YTH-expressing Neuro2a, NIH3T3 and mHippoE-2 cells. *n* = 3 for all conditions. (**C**) Representative images of DART-FISH targeting *Actb* m^6^A1277 in the long *Actb* isoform in the indicated cell lines. Dotted lines represent cell outlines. Scale bars = 10 μm. (**D**) Quantification of the total number of long isoform *Actb* RCPs detected by DART-FISH in individual cells for each indicated cell type. Solid line represents median with quartiles shown by dotted lines. Neuro2a: = 115, NIH3T3: *n* = 90; mHippoE-2: *n* = 22. ****P < 0.0001, ns = not statistically significant. (**E**) Quantification of the percentage of transcripts with m^6^A1277 detected by DART-FISH in individual cells for the indicated cell types. Solid line represents median with quartiles shown by dotted lines. Neuro2a: *n* = 115; NIH3T3: *n* = 90; mHippoE-2: *n* = 22; ****P < 0.0001, ***P < 0.001. (**F**) Quantification of %C2U adjacent to *Actb* A1277 determined by 3′RACE coupled with PCR and sequencing of the long isoform (left) or short isoform (right) in the three cell types. Values shown are means ± s.d.; *n* = 3 for all conditions. ****P < 0.0001, ***P < 0.001, ns = not statistically significant.

Since DART-FISH incorporates a reverse transcription (RT) step utilizing a primer specific to a target RNA of interest, we reasoned that we could interrogate m^6^A in specific transcript variants by designing isoform-specific RT primers (29). To test this, we chose an m^6^A site within the mouse *Actb* 3′UTR at A1277, which is 19 nucleotides upstream of an alternative polyadenylation (APA) site (30,31). Usage of this upstream polyadenylation site produces a short transcript isoform with a truncated (60 nt) 3′UTR, whereas usage of a downstream polyadenylation site produces a long isoform with a full-length (1916 nt) 3′UTR (Figure 4A) (30).

To test isoform-specific m^6^A detection, we used DART-FISH to target *Actb* A1277 in three mouse cell lines of different origins: NIH3T3 (embryonic fibroblast cells), Neuro2a (neuroblast cells), and mHippoE-2 (embryonic hippocampal neuronal cells). We generated stable cells expressing inducible APO1-YTH for each cell line and confirmed APO1-YTH expression and editing (Supplementary Figure S4). We then confirmed the expression of both long and short *Actb* isoforms in all three cell lines using 3′rapid amplification of cDNA ends (3′RACE) (Figure 4B).

Next, we designed an RT primer which hybridizes downstream of the first APA site, enabling DART-FISH detection of A/m^6^A1277 specifically in the long 3′UTR isoform (Figure 4A). DART-FISH confirmed the presence of the long *Actb* isoform in all three cell lines and revealed that *Actb* A1277 methylation in the long isoform is variable among the three cell types (Figure 4C–E). In addition, consistent with scDART-seq data (19) and with what we observed in HEK293T cells for the *HNRNPA1* and *EEF1A1* transcripts, the methylation level at *Actb* A1277 is variable between individual cells for all three cell types, ranging from 0–54.7% in Neuro2a cells, 0–23.4% in NIH3T3 cells and 0–31.8% in mHippoE-2 cells (Figure 4E).

Due to the close proximity of A1277 to the upstream APA site, it is not possible to target the short *Actb* isoform with DART-FISH probes (Figure 4A). However, using 3′ RACE followed by short and long *Actb* isoform amplification and sequencing, we quantified isoform-specific levels of A1277 methylation by measuring C-to-U editing at C1278 (Figure 4F). This confirmed the variable methylation levels of A1277 across the three cell types that we observed with DART-FISH (Figure 4C-E). Additionally, this revealed similar levels of A1277 methylation in the short and long isoforms within each cell line. For instance, in Neuro2a cells, the short and long isoforms have C2U values of 24% and 28%, respectively. Similar results were observed in NIH3T3 cells (short: 6%; long: 11%) and mHippoE-2 cells (short: 11%, long: 14%) (Figure 4F). Altogether, these data demonstrate that DART-FISH can visualize m^6^A in distinct transcript isoforms and can be used to reveal differential methylation in transcript variants across cells.

### m^6^A is not sufficient for recruitment of mRNAs to stress granules

Previous studies have suggested that m^6^A promotes mRNA localization to stress granules (SGs) through recruitment of YTHDF proteins and that this effect is most pronounced for RNAs with multiple m^6^A sites (5,6,8). However, other studies have suggested that m^6^A plays a very limited role in mRNA recruitment to SGs (7). A major limitation for studying the effects of m^6^A on the localization of mRNAs to SGs has been the lack of methods that assess the methylation state of transcripts recruited to SGs. Since DART-FISH enables simultaneous visualization of m^6^A-modified and unmodified transcripts in cells, we reasoned that we could use this method to investigate the partitioning of methylated mRNAs to SGs relative to their unmethylated counterparts.

For these studies, we used U-2 OS cells expressing the SG marker G3BP1 fused to GFP (32,33) in which we stably integrated doxycycline-inducible APO1-YTH (Supplementary Figure S5A). Treatment with 500μM sodium arsenite for 1h led to robust SG formation which was not influenced by the presence of APO1-YTH (Supplementary Figure S5B–E). To investigate the localization of methylated and unmethylated transcripts to SGs, we subjected arsenite-treated cells to DART-FISH followed by super-resolution microscopy to measure the colocalization of m^6^A-modified and unmodified transcripts with G3BP1-marked SGs. We focused on the *ACTB* and *HNRNPA1* mRNAs due to their high expression level and the fact that they both contain sites with relatively high levels of m^6^A.

**Figure 5.**
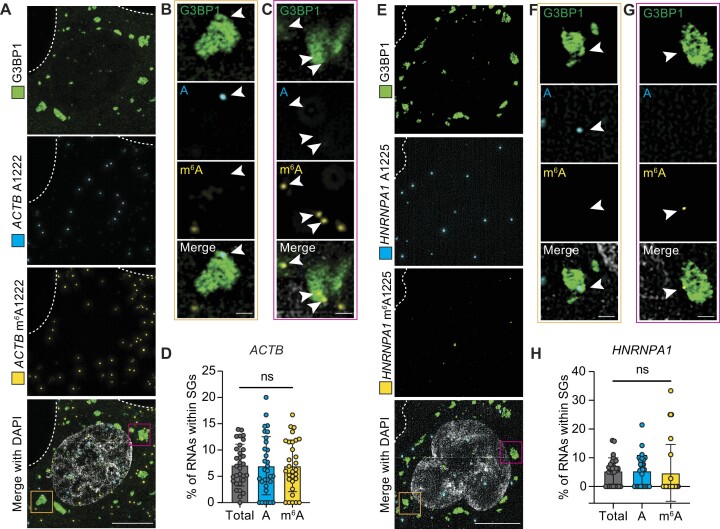
m^6^A is not sufficient for mRNA recruitment to stress granules. (**A**) Representative images of APO1-YTH-expressing U-2 OS G3BP1-GFP cells treated with 500 μM sodium arsenite for 1 h. DART-FISH was performed targeting the *ACTB* m^6^A1222 site followed by immunofluorescence to detect G3BP1. Dotted lines represent cell outline. Scale bars = 10 μm. (**B**) High-magnification images of a single z-plane of the orange boxed region in (A). Arrows indicate stress granule colocalized RCPs. Scale bar = 1 μm. (**C**) High-magnification images of a single z-plane of the red boxed region in (A). Arrows indicate stress granule colocalized RCPs. Scale bar = 1 μm. (**D**) Quantification of the methylated, unmethylated, and total number of *ACTB* A1222 RCPs colocalized with stress granules in individual U-2 OS cells after sodium arsenite treatment. Data points represent individual cells. Values shown are means ± s.d. *n* = 31 for all conditions. ns = not statistically significant. (**E**) Representative images of APO1-YTH-expressing U-2 OS G3BP1-GFP cells treated with 500 μM sodium arsenite for 1 h. DART-FISH was performed targeting the *HNRNPA1* m^6^A1225 site followed by immunofluorescence to detect G3BP1. Dotted lines represent cell outline. Scale bars = 10 μm. (**F**) High-magnification images of a single z-plane of the orange boxed region in (E). Arrows indicate stress granule colocalized RCPs. Scale bar = 1 μm. (**G**) High-magnification images of a single z-plane of the red boxed region in (E). Arrows indicate stress granule colocalized RCPs. Scale bar = 1μm. (**H**) Quantification of the methylated, unmethylated, and total number of *HNRNPA1* A1225 RCPs colocalized with stress granules in individual U-2 OS cells after sodium arsenite treatment. Data points represent individual cells. Values shown are means ± s.d. *n* = 29 for all conditions. ns = not statistically significant.

First, we assessed the overall localization of *ACTB* and *HNRNPA1* to SGs. We found that 7.1% of *ACTB* transcripts and 5.4% of *HNRNPA1* transcripts colocalize with SGs, which is similar to what has been previously reported using SG purification and RNA-seq (Figure 5) (33,34). We further validated this using smFISH targeting *ACTB*, which indicated a similar proportion of transcripts localizing to SGs as we observed with DART-FISH (Supplementary Figure S5F, G). We next examined the localization of m^6^A-modified and unmodified transcripts to SGs. We focused on *ACTB* A1222 and *HNRNPA1* A1225 since these sites are methylated with high frequency and are therefore likely to have greater potential for influencing transcript localization. Surprisingly, we found nearly identical SG localization frequencies of methylated and unmethylated transcripts for both mRNAs. For *ACTB*, 7% of A1222 and 6.99% of m^6^A1222 transcripts localize to SGs (Figure 5A-D), and for *HNRNPA1*, we observed 5.49% of A1225 and 4.79% of m^6^A1225 transcripts localized to SGs (Figure 5E–H). Thus, these data indicate that m^6^A-modified transcripts are not preferentially localized to SGs, at least for the sites and transcripts tested here.

**Figure 6.**
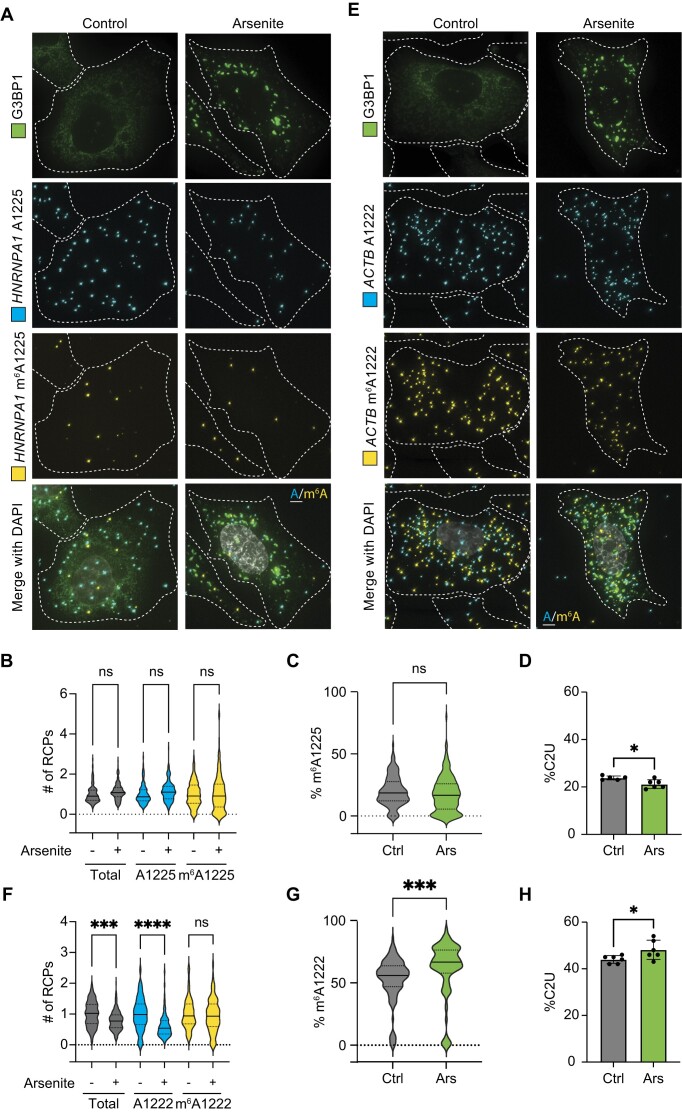
Visualization of m^6^A-modified mRNAs following oxidative stress. (**A**) Representative DART-FISH images targeting *HNRNPA1* m^6^A1225 in U-2 OS G3BP1-GFP cells treated for 1h with water or 500μM sodium arsenite. G3BP1 was detected by immunofluorescence. Dotted lines represent cell outlines. Scale bars = 5 μm. (**B**) Quantification of the methylated, unmethylated, and total number of *HNRNPA1* A1225 RCPs in individual U-2 OS G3BP1-GFP cells treated with water or sodium arsenite. Values are plotted relative to the population average in control-treated cells. Solid line represents median with quartiles shown by dotted lines. Control: *n* = 145; arsenite: *n* = 171. ns = not statistically significant. (**C**) %C2U adjacent to *HNRNPA1* A1225 determined by DART-FISH in U-2 OS G3BP1-GFP cells treated with water or sodium arsenite. Solid line represents median with quartiles shown by dotted lines. Control: *n* = 145; Arsenite: *n* = 171. ns = not statistically significant. (**D**) %C2U adjacent to *HNRNPA1* A1225 determined by RT-PCR and Sanger sequencing of U-2 OS G3BP1-GFP cells treated with water or sodium arsenite. Values shown are means ± s.d. Control: *n* = 5; Arsenite: *n* = 6. *P < 0.05. (**E**) Representative DART-FISH images targeting *ACTB* m^6^A1222 in U-2 OS G3BP1-GFP cells treated for 1 h with water or 500 μM sodium arsenite. G3BP1 was detected by immunofluorescence. Dotted lines represent cell outlines. Scale bars = 5 μm. (**F**) Quantification of the methylated, unmethylated, and total number of *ACTB* A1222 RCPs in individual U-2 OS G3BP1-GFP cells treated with water or sodium arsenite. Values are plotted relative to the population average in control-treated cells. Solid line represents median with quartiles shown by dotted lines. Control: *n* = 118; Arsenite: *n* = 174. ****P < 0.0001, ***P < 0.001, ns = not statistically significant. (**G**) %C2U adjacent to *ACTB* A1222 determined by DART-FISH in U-2 OS G3BP1-GFP cells treated with water or sodium arsenite. Solid line represents median with quartiles shown by dotted lines. Control: *n* = 118; Arsenite: *n* = 174. ***P < 0.001. (**H**) %C2U adjacent to *ACTB* A1222 determined by RT-PCR and Sanger sequencing of U-2 OS G3BP1-GFP cells treated with water or sodium arsenite. Values shown are means ± s.d.; *n* = 6. *P < 0.05.

Previous studies have suggested dynamic regulation of m^6^A following oxidative stress, with some mRNAs exhibiting elevated m^6^A levels after sodium arsenite treatment (8). To determine whether the *ACTB* A1222 and *HNRNPA1* A1225 sites are dynamically regulated during stress, we used DART-FISH to quantify the number of methylated and unmethylated transcripts before and after sodium arenite treatment. For *HNRNPA1*, we found no significant change in the number of unmodified and m^6^A-modified A1225 transcripts after arsenite treatment (Figure 6A–D). However, for *ACTB*, we found static levels of m^6^A-modified transcripts but a selective reduction in unmodified A1222 transcripts, which resulted in fewer total transcripts being detected after stress (Figure 6E–G). These findings at the single-molecule level were also supported by RT-PCR/Sanger sequencing of bulk cells, which indicates an elevated %C2U value at *ACTB* C1223 after sodium arsenite treatment (Figure 6H). Together, these data demonstrate the ability of DART-FISH to detect stress-induced changes in the abundance of methylated and non-methylated transcripts.

## Discussion

A major limitation for m^6^A research has been the dearth of methods for visualizing methylated transcripts in cells. DART-FISH overcomes this challenge by enabling *in situ* detection of individual m^6^A sites in RNAs of interest. Our method is highly versatile and can be used to detect methylated mRNAs in a variety of cell types. Importantly, DART-FISH also enables simultaneous visualization of methylated and unmethylated transcripts in cells, which provides new insights into m^6^A stoichiometry and facilitates studies of m^6^A dynamics and methylated RNA localization.

To demonstrate the utility of DART-FISH, we used it to address recent questions that have emerged regarding the role of m^6^A in mRNA localization to SGs. RNA recruitment to SGs is an important regulatory mechanism for tuning gene expression responses during cellular stress (35). However, the mechanisms that control mRNA partitioning to SGs are not completely understood. While some studies have suggested that m^6^A promotes the localization of mRNAs to SGs (5,6,8), others have found that m^6^A plays only a very minor role (7). To address this discrepancy, we used DART-FISH to quantify m^6^A-modified and unmodified transcripts in SGs following oxidative stress. Our data suggest that m^6^A-modified mRNAs are not preferentially localized to SGs, indicating that m^6^A is not sufficient for SG recruitment for the few transcripts and m^6^A sites that we tested here. However, it remains possible that m^6^A sites in other mRNAs play a more dominant role in partitioning mRNAs to SGs. Future studies applying DART-FISH to additional transcripts and in response to other forms of cellular stress will be informative.

One point of debate within the m^6^A field has been the degree to which m^6^A is dynamically regulated in cells. We envision that DART-FISH will serve as a much-needed tool for investigating this question. While we applied DART-FISH here to examine changes in m^6^A stoichiometry and methylated mRNA localization following cellular stress, there are multiple scenarios in which DART-FISH can be utilized to address m^6^A dynamics. For instance, m^6^A levels in some transcripts undergo activity-dependent regulation in neurons (4,31,36,37), and hundreds of mRNAs are differentially localized to neuronal axons and dendrites in response to loss of METTL3 (4). Thus, using DART-FISH to examine m^6^A abundance and m^6^A-modified mRNA localization in neurons following synaptic activity can provide new insights into activity-dependent m^6^A dynamics and the role of m^6^A in regulating mRNA localization in neurons.

One limitation of DART-FISH is that it relies on expression of the APO1-YTH protein to target m^6^A-adjacent cytidines for deamination. Thus, factors that influence APO1-YTH expression or activity could compromise m^6^A detection. APO1-YTH expression is primarily cytoplasmic, and although nuclear-localized RNAs can be edited by the enzyme (18,19), its efficiency for labeling methylated RNAs may be improved by targeting it to the nucleus. Additionally, although one advantage of DART-FISH is its ability to detect m^6^A with single-nucleotide resolution, some sites may be inherently difficult to detect due to inaccessibility to APO1-YTH. DART-FISH is also subject to the same limitations inherent to padlock probe-based strategies for RNA detection, so some m^6^A sites may be challenging to target due to nearby sequence or structural elements. Compared to smFISH, these strategies generally detect less than 30% of transcript molecules in the cell (15,38), which is consistent with what we observe using DART-FISH (Supplementary Figure S5H), so only the relative number of methylated and unmethylated RNA molecules for a given RNA can be determined. In theory, DART-based editing can be combined with any SNV-sensitive RNA detection strategy, so other methods for RNA targeting and signal amplification can potentially be used instead of padlock probes and rolling circle amplification.

Here, we demonstrate the utility of DART-FISH for visualizing methylated RNAs using stable cell lines expressing APO1-YTH. However, strategies such as viral delivery or transgenic animal models could also be used to express the APO1-YTH protein. Such approaches would enable investigation of m^6^A in a broad range of tissues and cell types of interest and would be particularly useful for studying m^6^A *in vivo*. Overall, we expect that DART-FISH will open up several new avenues of m^6^A research, including the study of m^6^A dynamics in cells and investigations of the localization of methylated and non-methylated transcripts at the subcellular level.

## Supplementary Material

gkad787_Supplemental_Files

## Data Availability

Data supporting the findings of this study are available upon request due to the size and number of imaging datasets.

## References

[1] Jiang X. , Liu B., Nie Z., Duan L., Xiong Q., Jin Z., Yang C., Chen Y. The role of m6A modification in the biological functions and diseases. Signal Transduct. Target Ther. 2021; 6:74. 33611339 10.1038/s41392-020-00450-x PMC7897327

[2] Zaccara S. , Ries R.J., Jaffrey S.R. Reading, writing and erasing mRNA methylation. Nat. Rev. Mol. Cell Biol. 2019; 20:608–624. 31520073 10.1038/s41580-019-0168-5

[3] Roundtree I.A. , Luo G.-Z., Zhang Z., Wang X., Zhou T., Cui Y., Sha J., Huang X., Guerrero L., Xie P. et al. YTHDC1 mediates nuclear export of N^6^-methyladenosine methylated mRNAs. eLife. 2017; 6:e31311. 28984244 10.7554/eLife.31311 PMC5648532

[4] Flamand M.N. , Meyer K.D. m6A and YTHDF proteins contribute to the localization of select neuronal mRNAs. Nucleic Acids Res. 2022; 50:4464–4483. 35438793 10.1093/nar/gkac251 PMC9071445

[5] Ries R.J. , Pickering B.F., Poh H.X., Namkoong S., Jaffrey S.R. m^6^A governs length-dependent enrichment of mRNAs in stress granules. Nat. Struct. Mol. Biol. 2022; 10.1038/s41594-023-01089-2. PMC10715973 37710015

[6] Fu Y. , Zhuang X. m(6)A-binding YTHDF proteins promote stress granule formation. Nat. Chem. Biol. 2020; 16:955–963. 32451507 10.1038/s41589-020-0524-y PMC7442727

[7] Khong A. , Matheny T., Huynh T.N., Babl V., Parker R. Limited effects of m(6)A modification on mRNA partitioning into stress granules. Nat. Commun. 2022; 13:3735. 35768440 10.1038/s41467-022-31358-5 PMC9243116

[8] Anders M. , Chelysheva I., Goebel I., Trenkner T., Zhou J., Mao Y., Verzini S., Qian S.B., Ignatova Z. Dynamic m(6)A methylation facilitates mRNA triaging to stress granules. Life Sci. Alliance. 2018; 1:e201800113. 30456371 10.26508/lsa.201800113 PMC6238392

[9] Gasparski A.N. , Mason D.E., Moissoglu K., Mili S. Regulation and outcomes of localized RNA translation. Wiley Interdiscip. Rev. RNA. 2022; 13:e1721. 35166036 10.1002/wrna.1721 PMC9787767

[10] Ren X. , Deng R., Zhang K., Sun Y., Li Y., Li J. Single-cell imaging of m(6) a modified RNA using m(6) a-specific in situ hybridization mediated proximity ligation assay (m(6) AISH-PLA). Angew. Chem. Int. Ed. Engl. 2021; 60:22646–22651. 34291539 10.1002/anie.202109118

[11] Zhao X. , Ji X., Qu J., Xie K., Wang Z., Fang P., Wang Y., Wan Y., Yang Y., Zhang W. et al. Sequencing-free analysis of multiple methylations on gene-specific mRNAs. J. Am. Chem. Soc. 2022; 144:6010–6018. 35321539 10.1021/jacs.2c01036

[12] Kim K.L. , van Galen P., Hovestadt V., Rahme G.J., Andreishcheva E.N., Shinde A., Gaskell E., Jones D.R., Shema E., Bernstein B.E. Systematic detection of m(6)A-modified transcripts at single-molecule and single-cell resolution. Cell Rep. Methods. 2021; 1:100061. 34734208 10.1016/j.crmeth.2021.100061 PMC8562683

[13] Le P. , Ahmed N., Yeo G.W. Illuminating RNA biology through imaging. Nat. Cell Biol. 2022; 24:815–824. 35697782 10.1038/s41556-022-00933-9 PMC11132331

[14] Levesque M.J. , Ginart P., Wei Y., Raj A. Visualizing SNVs to quantify allele-specific expression in single cells. Nat. Methods. 2013; 10:865–867. 23913259 10.1038/nmeth.2589 PMC3771873

[15] Larsson C. , Grundberg I., Soderberg O., Nilsson M. In situ detection and genotyping of individual mRNA molecules. Nat. Methods. 2010; 7:395–397. 20383134 10.1038/nmeth.1448

[16] Mellis I.A. , Gupte R., Raj A., Rouhanifard S.H. Visualizing adenosine-to-inosine RNA editing in single mammalian cells. Nat. Methods. 2017; 14:801–804. 28604724 10.1038/nmeth.4332 PMC5542810

[17] Lundin E. , Wu C., Widmark A., Behm M., Hjerling-Leffler J., Daniel C., Ohman M., Nilsson M. Spatiotemporal mapping of RNA editing in the developing mouse brain using in situ sequencing reveals regional and cell-type-specific regulation. BMC Biol. 2020; 18:6. 31937309 10.1186/s12915-019-0736-3 PMC6961268

[18] Meyer K.D. DART-seq: an antibody-free method for global m(6)A detection. Nat. Methods. 2019; 16:1275–1280. 31548708 10.1038/s41592-019-0570-0 PMC6884681

[19] Tegowski M. , Flamand M.N., Meyer K.D. scDART-seq reveals distinct m(6)A signatures and mRNA methylation heterogeneity in single cells. Mol. Cell. 2022; 82:868–878. 35081365 10.1016/j.molcel.2021.12.038 PMC8857065

[20] Schindelin J. , Arganda-Carreras I., Frise E., Kaynig V., Longair M., Pietzsch T., Preibisch S., Rueden C., Saalfeld S., Schmid B. et al. Fiji: an open-source platform for biological-image analysis. Nat. Methods. 2012; 9:676–682. 22743772 10.1038/nmeth.2019 PMC3855844

[21] Kluesner M.G. , Nedveck D.A., Lahr W.S., Garbe J.R., Abrahante J.E., Webber B.R., Moriarity B.S. EditR: a method to quantify base editing from sanger sequencing. CRISPR J. 2018; 1:239–250. 31021262 10.1089/crispr.2018.0014 PMC6694769

[22] Castellanos-Rubio A. , Santin I., Olazagoitia-Garmendia A., Romero-Garmendia I., Jauregi-Miguel A., Legarda M., Bilbao J.R. A novel RT-QPCR-based assay for the relative quantification of residue specific m6A RNA methylation. Sci. Rep. 2019; 9:4220. 30862814 10.1038/s41598-019-40018-6 PMC6414506

[23] Carpenter A.E. , Jones T.R., Lamprecht M.R., Clarke C., Kang I.H., Friman O., Guertin D.A., Chang J.H., Lindquist R.A., Moffat J. et al. CellProfiler: image analysis software for identifying and quantifying cell phenotypes. Genome Biol. 2006; 7:R100. 17076895 10.1186/gb-2006-7-10-r100 PMC1794559

[24] Tsanov N. , Samacoits A., Chouaib R., Traboulsi A.M., Gostan T., Weber C., Zimmer C., Zibara K., Walter T., Peter M. et al. smiFISH and FISH-quant - a flexible single RNA detection approach with super-resolution capability. Nucleic Acids Res. 2016; 44:e165. 27599845 10.1093/nar/gkw784 PMC5159540

[25] Linder B. , Grozhik A.V., Olarerin-George A.O., Meydan C., Mason C.E., Jaffrey S.R. Single-nucleotide-resolution mapping of m6A and m6Am throughout the transcriptome. Nat. Methods. 2015; 12:767–772. 26121403 10.1038/nmeth.3453 PMC4487409

[26] Liu C. , Sun H., Yi Y., Shen W., Li K., Xiao Y., Li F., Li Y., Hou Y., Lu B. et al. Absolute quantification of single-base m^6^A methylation in the mammalian transcriptome using GLORI. Nat. Biotechnol. 2023; 41:355–366. 36302990 10.1038/s41587-022-01487-9

[27] Zhu H. , Yin X., Holley C.L., Meyer K.D. Improved methods for deamination-based m(6)A detection. Front. Cell Dev. Biol. 2022; 10:888279. 35573664 10.3389/fcell.2022.888279 PMC9092492

[28] Yankova E. , Blackaby W., Albertella M., Rak J., De Braekeleer E., Tsagkogeorga G., Pilka E.S., Aspris D., Leggate D., Hendrick A.G. et al. Small-molecule inhibition of METTL3 as a strategy against myeloid leukaemia. Nature. 2021; 593:597–601. 33902106 10.1038/s41586-021-03536-w PMC7613134

[29] Ren X. , Deng R., Zhang K., Sun Y., Teng X., Li J. SpliceRCA: in situ single-cell analysis of mRNA splicing variants. ACS Cent Sci. 2018; 4:680–687. 29974063 10.1021/acscentsci.8b00081 PMC6026782

[30] Ghosh T. , Soni K., Scaria V., Halimani M., Bhattacharjee C., Pillai B. MicroRNA-mediated up-regulation of an alternatively polyadenylated variant of the mouse cytoplasmic beta-actin gene. Nucleic Acids Res. 2008; 36:6318–6332. 18835850 10.1093/nar/gkn624 PMC2577349

[31] Weng Y.L. , Wang X., An R., Cassin J., Vissers C., Liu Y., Liu Y., Xu T., Wang X., Wong S.Z.H. et al. Epitranscriptomic m(6)A regulation of axon regeneration in the adult mammalian nervous system. Neuron. 2018; 97:313–325. 29346752 10.1016/j.neuron.2017.12.036 PMC5777326

[32] Figley M.D. , Bieri G., Kolaitis R.M., Taylor J.P., Gitler A.D. Profilin 1 associates with stress granules and ALS-linked mutations alter stress granule dynamics. J. Neurosci. 2014; 34:8083–8097. 24920614 10.1523/JNEUROSCI.0543-14.2014 PMC4051967

[33] Khong A. , Matheny T., Jain S., Mitchell S.F., Wheeler J.R., Parker R. The stress granule transcriptome reveals principles of mRNA accumulation in stress granules. Mol. Cell. 2017; 68:808–820. 29129640 10.1016/j.molcel.2017.10.015 PMC5728175

[34] Namkoong S. , Ho A., Woo Y.M., Kwak H., Lee J.H. Systematic characterization of stress-induced RNA granulation. Mol. Cell. 2018; 70:175–187. 29576526 10.1016/j.molcel.2018.02.025 PMC6359928

[35] Campos-Melo D. , Hawley Z.C.E., Droppelmann C.A., Strong M.J. The integral role of RNA in stress granule formation and function. Front. Cell Dev. Biol. 2021; 9:621779. 34095105 10.3389/fcell.2021.621779 PMC8173143

[36] Widagdo J. , Zhao Q.Y., Kempen M.J., Tan M.C., Ratnu V.S., Wei W., Leighton L., Spadaro P.A., Edson J., Anggono V. et al. Experience-dependent accumulation of N6-methyladenosine in the prefrontal cortex is associated with memory processes in mice. J. Neurosci. 2016; 36:6771–6777. 27335407 10.1523/JNEUROSCI.4053-15.2016 PMC4916251

[37] Zhang Z. , Wang M., Xie D., Huang Z., Zhang L., Yang Y., Ma D., Li W., Zhou Q., Yang Y.G. et al. METTL3-mediated N(6)-methyladenosine mRNA modification enhances long-term memory consolidation. Cell Res. 2018; 28:1050–1061. 30297870 10.1038/s41422-018-0092-9 PMC6218447

[38] Krzywkowski T. , Nilsson M. Padlock probes to detect single nucleotide polymorphisms. Methods Mol. Biol. 2018; 1649:209–229. 29130200 10.1007/978-1-4939-7213-5_14

