# Supplementary material for: In situ visualization of m6A sites in cellular mRNAs
Source: Nucleic Acids Res. 2023 Oct 9;51(20):e101. doi: 10.1093/nar/gkad787 (PMC10639046; doi:10.1093/nar/gkad787)
Supplement: gkad787_Supplemental_Files [file gkad787_supplemental_files.zip › Supplementary Material.pdf]

Supplementary Table 1		
**Oligonucleotide Name**	**Sequence (5' - 3')**	**Purpose**
ACTB 5’UTR Fwd	CACCACCACCGCCGAGACCGCG	Cloning pCMV-mNeonGreen-ACTB-C1223
ACTB 5’UTR Rev	CTCACCATGGTGAGCTGGCG	Cloning pCMV-mNeonGreen-ACTB-C1223
mNeonGreen Fwd	AGCTCACCATGGTGAGTAAGGGCGAAGAG	Cloning pCMV-mNeonGreen-ACTB-C1223
mNeonGreen Rev	CGATATCATCATCTTTATACAACTCGTCCATGCCCATCAC	Cloning pCMV-mNeonGreen-ACTB-C1223
ACTB CDS + 3’ UTR Fwd	GAGTTGTATAAAGATGATGATATCGCCGCGCTCGT	Cloning pCMV-mNeonGreen-ACTB-C1223
ACTB CDS + 3’ UTR Rev	TCGGGTTTAAACTCATTTTTAAGGTGTGCACTTTTATTCAACTGG	Cloning pCMV-mNeonGreen-ACTB-C1223
pCMV Vector Fwd	CTTAAAAATGAGTTTAAACCCGAAACTTAAACCCG	Cloning pCMV-mNeonGreen-ACTB-C1223
pCMV Vector Rev	TCGGCGGTGGTGGTGGCGGCTCT	Cloning pCMV-mNeonGreen-ACTB-C1223
ACTB U1223 Fwd	GCTTCTAGGCGGACTATGATTTAGTTGCGTTACAC	Cloning pCMV-mNeonGreen-ACTB-U1223
ACTB U1223 Vector Rev	TAGTCCGCCTAGAAGCATTTGC	Cloning pCMV-mNeonGreen-ACTB-U1223
APO1-YTH_F	ggttctagagcgctgccaccatgagctcagag	Cloning TLCV2-APO1-YTH
APO1-YTH_R	cgctctgcccggatgctagctcaggcgtagtcgggca	Cloning TLCV2-APO1-YTH
TLCV2_F	ggttctagagcgctgccaccatgagctcagag	Cloning TLCV2-APO1-YTH
TLCV2_R	ggttctagagcgctgccaccatgagctcagag	Cloning TLCV2-APO1-YTH
ACTB_F	GATCATTGCTCCTCCTGAGCGCAAG	ACTB A1222 RT-PCR and Sanger sequencing
ACTB_R	GCCATGCCAATCTCATCTTGTTTTCTG	ACTB A1222 RT-PCR and Sanger sequencing
HNRNPA1_F	cagcagcagcagtagctatgg	HNRNPA1 A1225 RT-PCR and Sanger sequencing
HNRNPA1_R	cctttgttggaatgctttacactttcc	HNRNPA1 A1225 RT-PCR and Sanger sequencing
3´RACE_oligodT-adapter	GGCCACGCGTCGACTAGTAC (T)17	3´ RACE
3´RACE_adapter_R	GGCCACGCGTCGACTAGTAC	3´ RACE RT-PCR
mActb_3'RACE_F	GGCATTGCTGACAGGATGCAGAAGG	mActb 3´RACE RT-PCR
mActb_3'RACE_nested_F	gaaggagattactgctctggctcc	mActb 3´RACE RT-PCR
mActb_3´RACE_long_F	ttccagcagatgtggatcag	mActb 3´RACE RT-PCR
mHnrnpa1_F	gtggtggacagggttatggaaacc	Mouse Hnrnpa1 RT-PCR and Sanger sequencing
mHnrnpa1_R	attccaaatggaacttggcaccacc	Mouse Hnrnpa1 RT-PCR and Sanger sequencing
mAkt1_F	caggtcaggaggaaaactatcctgg	Mouse Akt1 RT-PCR and Sanger sequencing
mAkt1_R	ccagacccatgagcccacattg	Mouse Akt1 RT-PCR and Sanger sequencing
ACTB_A1222_Adj(+)	ttgtcaagaaagggtgtaacgcaactaag	ACTB A1222 SELECT and RT-qPCR
ACTB_A1222_Non-Adj (-)	catgccaatctcatcttg	ACTB A1222 SELECT
ACTB_A1222_qPCR_F	cagcaagcaggagtatgacgagtc	ACTB A1222 SELECT RT-qPCR
ACTB_A947_Non-adj (-)	cacgtcacacttcatgatggagttg	ACTB A947 SELECT and RT-qPCR
ACTB_A947_Adj (+)	gcgtacaggtctttgcggatg	ACTB A947 SELECT
ACTB_A947_qPCR_F	ggtcatcaccattggcaatgagc	ACTB A947 SELECT RT-qPCR
P-ACTB-A1222	G+CC +AT+G C+CA +AT+C T+CA TCT TGT TTT CTG	ACTB A1222 DART-FISH
PLP-ACTB-U1223-DT2	/5Phos/TTAGTTGCGTTACACCCTTTCAAAAAAAAAAAAAAACCTCAATGCACATGTTTGGCTCCAAAAAAAAAAAAAAACTTCTAGGCGGACTATGAT	ACTB A1222 DART-FISH
PLP-ACTB-C1223-DT3	/5Phos/TTAGTTGCGTTACACCCTTTCAAAAAAAAAAAAAAAAAGTAGCCGTGACTATCGACTAAAAAAAAAAAAAAAACTTCTAGGCGGACTATGAC	ACTB A1222 DART-FISH
P-mActb-A1277	+aa+aa+ac+aa+ag+cc+atgccaat	mActb A1277 DART-FISH
PLP-mActb-C1278-DT3	/5Phos/AAAACCTAACTTGCGCAGAAAAAAAAAAAAAAAAAGTAGCCGTGACTATCGACTAAAAAAAAAAAAAACGTTTTACACCCTTTCTTTGAC	mActb A1277 DART-FISH
PLP-mActb-U1278-DT4	/5Phos/AAAACCTAACTTGCGCAGAAAAAAAAAAAAAAAAATGCGTCTATTTAGTGGAGCCAAAAAAAAAAAAAACGTTTTACACCCTTTCTTTGAT	mActb A1277 DART-FISH
P-HNRNPA1-A1225	C+CC +TT+T G+TT+ G+GA+ATG CTT TAC ACT TTC C	HNRNPA1 A1225 DART-FISH
PLP-HNRNPA1-C1226–DT2	/5Phos/TAATTGTATAACAGGTTATTAAAAAAAAAAAAAACCTCAATGCACATGTTTGGCTCCAAAAAAAAAAAAAATCGAGGACTGTATTTGTGAC	HNRNPA1 A1225 DART-FISH
PLP-HNRNPA1-U1226–DT3	/5Phos/TAATTGTATAACAGGTTATTAAAAAAAAAAAAAAAAGTAGCCGTGACTATCGACTAAAAAAAAAAAAAAATCGAGGACTGTATTTGTGAT	HNRNPA1 A1225 DART-FISH
P-EEF1A1-A167	c+ct+tt+cc+ca+tc+tc+agcagcctcc	EEF1A1 A167 DART-FISH
PLP-EEF1A1-C168 – DT3	/5Phos/aaaagaaccattgaaaaattt AAA AAA AAA AAA AAA AGT AGC CGT GAC TAT CGA CT AAA AAA AAA AAA AAA taaatgcggtggcatcgac	EEF1A1 A167 DART-FISH
PLP-EEF1A1-U168 – DT4	/5Phos/aaaagaaccattgaaaaatttAAAAAAAAAAAAAAATGCGTCTATTTAGTGGAGCCAAAAAAAAAAAAAAA taaatgcggtggcatcgat	EEF1A1 A167 DART-FISH
DT4-CY3	/5Cy3/TGCGTCTATTTAGTGGAGCC	DART-FISH Detection
DT2-Cy5	/5Cy5/CCTCAATGCACATGTTTGGCTCC	DART-FISH Detection
DT3-Cy3	/5Cy3/AGTAGCCGTGACTATCGACT	DART-FISH Detection
DT3-CY5	/5Cy5/AGTAGCCGTGACTATCGACT	DART-FISH Detection

DT2-CY3	/5Cy3/CCTCAATGCACATGTTTGGCTCC	DART-FISH Detection
ACTB_smFISH_1	cgatatcatcatccatggtg	ACTB smFISH
ACTB_smFISH_2	cacgatggaggggaagacgg	ACTB smFISH
ACTB_smFISH_3	acataggaatccttctgacc	ACTB smFISH
ACTB_smFISH_4	ggtacttcagggtgaggatg	ACTB smFISH
ACTB_smFISH_5	cagattttctccatgtcgtc	ACTB smFISH
ACTB_smFISH_6	acacgcagctcattgtagaa	ACTB smFISH
ACTB_smFISH_7	acatgatctgggtcatcttc	ACTB smFISH
ACTB_smFISH_8	ggatagcacagcctggatag	ACTB smFISH
ACTB_smFISH_9	catcacgatgccagtggtac	ACTB smFISH
ACTB_smFISH_10	tcgtagatgggcacagtgtg	ACTB smFISH
ACTB_smFISH_11	tcttcatgaggtagtcagtc	ACTB smFISH
ACTB_smFISH_12	taatgtcacgcacgatttcc	ACTB smFISH
ACTB_smFISH_13	atctcttgctcgaagtccag	ACTB smFISH
ACTB_smFISH_14	cagggaggagctggaagcag	ACTB smFISH
ACTB_smFISH_15	tcattgccaatggtgatgac	ACTB smFISH
ACTB_smFISH_16	gaaggtagtttcgtggatgc	ACTB smFISH
ACTB_smFISH_17	cgtcacacttcatgatggag	ACTB smFISH
ACTB_smFISH_18	tacaggtctttgcggatgtc	ACTB smFISH
ACTB_smFISH_19	caatgccagggtacatggtg	ACTB smFISH
ACTB_smFISH_20	atcttcattgtgctgggtgc	ACTB smFISH
ACTB_smFISH_21	ctcaggaggagcaatgatct	ACTB smFISH
ACTB_smFISH_22	cgatccacacggagtacttg	ACTB smFISH
ACTB_smFISH_23	tcatactcctgcttgctgat	ACTB smFISH
ACTB_smFISH_24	atttgcggtggacgatggag	ACTB smFISH
ACTB_smFISH_25	aagtcatagtccgcctagaa	ACTB smFISH
ACTB_smFISH_26	gtcaagaaagggtgtaacgc	ACTB smFISH
ACTB_smFISH_27	ttttctgcgcaagttaggtt	ACTB smFISH
ACTB_smFISH_28	cattgtgaactttgggggat	ACTB smFISH
ACTB_smFISH_29	gtgcaatcaaagtcctcggc	ACTB smFISH
ACTB_smFISH_30	cctgtaacaacgcatctcat	ACTB smFISH
ACTB_smFISH_31	cttttaggatggcaagggac	ACTB smFISH
ACTB_smFISH_32	tctccttagagagaagtggg	ACTB smFISH
ACTB_smFISH_33	gtggacttgggagaggactg	ACTB smFISH
ACTB_smFISH_34	aaagcaatgctatcacctcc	ACTB smFISH
ACTB_smFISH_35	aaaaaaggggggaagggggggg	ACTB smFISH
ACTB_smFISH_36	catacatctcaagttggggg	ACTB smFISH
ACTB_smFISH_37	actcccagggagaccaaaag	ACTB smFISH
ACTB_smFISH_38	gtctcaagtcagtgtacagg	ACTB smFISH
ACTB_smFISH_39	ggtgtgcacttttattcaac	ACTB smFISH

Supplementary Table 2

Hg38

Chr	Start	End	Strand	Site
7	5527735	5527736	-	ACTB_A1222
12	54284724	54284725	+	HNRNPA1_A1225
6	73519921	73519922	-	EEF1A1_A167

mm10

Chr	Start	End	Strand	Gene
5	142,903,757	142,903,758	-	Actb_A1277

Supplementary Figure 1

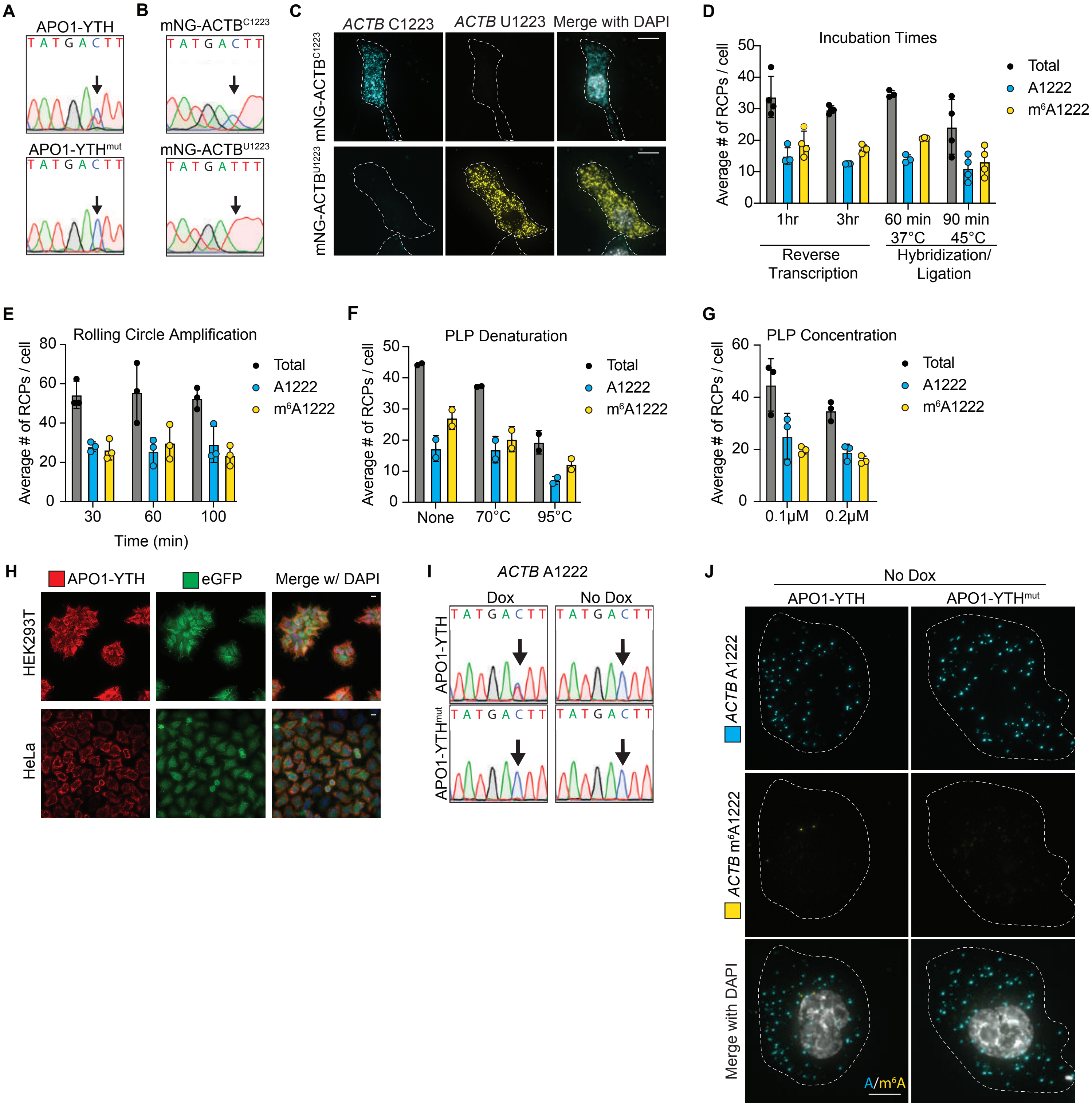

Supplementary Figure 1. Optimization of DART-FISH parameters.

(**A**) Representative RT-PCR/Sanger sequencing traces of the region surrounding *ACTB* A1222 in APO1-YTH- and APO1-YTH^{mut}-expressing HEK293T cells. Black arrow indicates C1223, the base edited adjacent to *ACTB* A1222. (**B**) Representative Sanger sequencing traces of mNeonGreen-ACTB^{C1223} and mNeonGreen-ACTB^{U1223} expression vectors confirming C or U mutation adjacent to *ACTB* A1222. Black arrow indicates position 1223. (**C**) Representative images of DART-FISH targeting *ACTB* m^{6}A1222 using C1223 or U1223 probes in NIH3T3 cells overexpressing either mNG-ACTB^{C1223} (top row) or mNG-ACTB^{U1223} (bottom row). Dotted line represents cell outline. Scale bars = 10µm. **D-G**, Quantification of the average number of RCPs per cell following DART-FISH targeting endogenous *ACTB* m^{6}A1222 in APO1-YTH-expressing HEK293T cells. Various conditions were tested: reverse transcription and hybridization/ligation times (**D**) (60 min at 37°C: n = 3; all other conditions: n = 4); rolling-circle amplification (**E**) (n = 3 for all conditions); padlock probe denaturation (**F**) (n = 2 for all conditions); and padlock probe concentration (**G**) (n = 3 for all conditions). Values plotted are mean ± s.d. Each graph shows the average number of total RCPs (left), A1222 RCPs (middle), or m^{6}A1222 (right) RCPs per cell. (**H**) Representative images of APO1-YTH-expressing HEK293T and HeLa cells following doxycycline induction of APO1-YTH-T2A-eGFP. Scale bar = 10µm. (**I**) Representative Sanger sequencing traces of the region surrounding *ACTB* A1222 in HeLa cells expressing APO1-YTH (top) or APO1-YTH^{mut} (bottom) with or without doxycycline induction. Black arrow represents *ACTB* C1223, the base edited adjacent to *ACTB* A1222. (**J**) Representative images of DART-FISH targeting *ACTB* m^{6}A1222 in HeLa cells expressing APO1-YTH (left) or APO1-YTH^{mut} (right) without doxycycline induction. Dotted lines represent cell outline and scale bar = 10µm.

Supplementary Figure 2

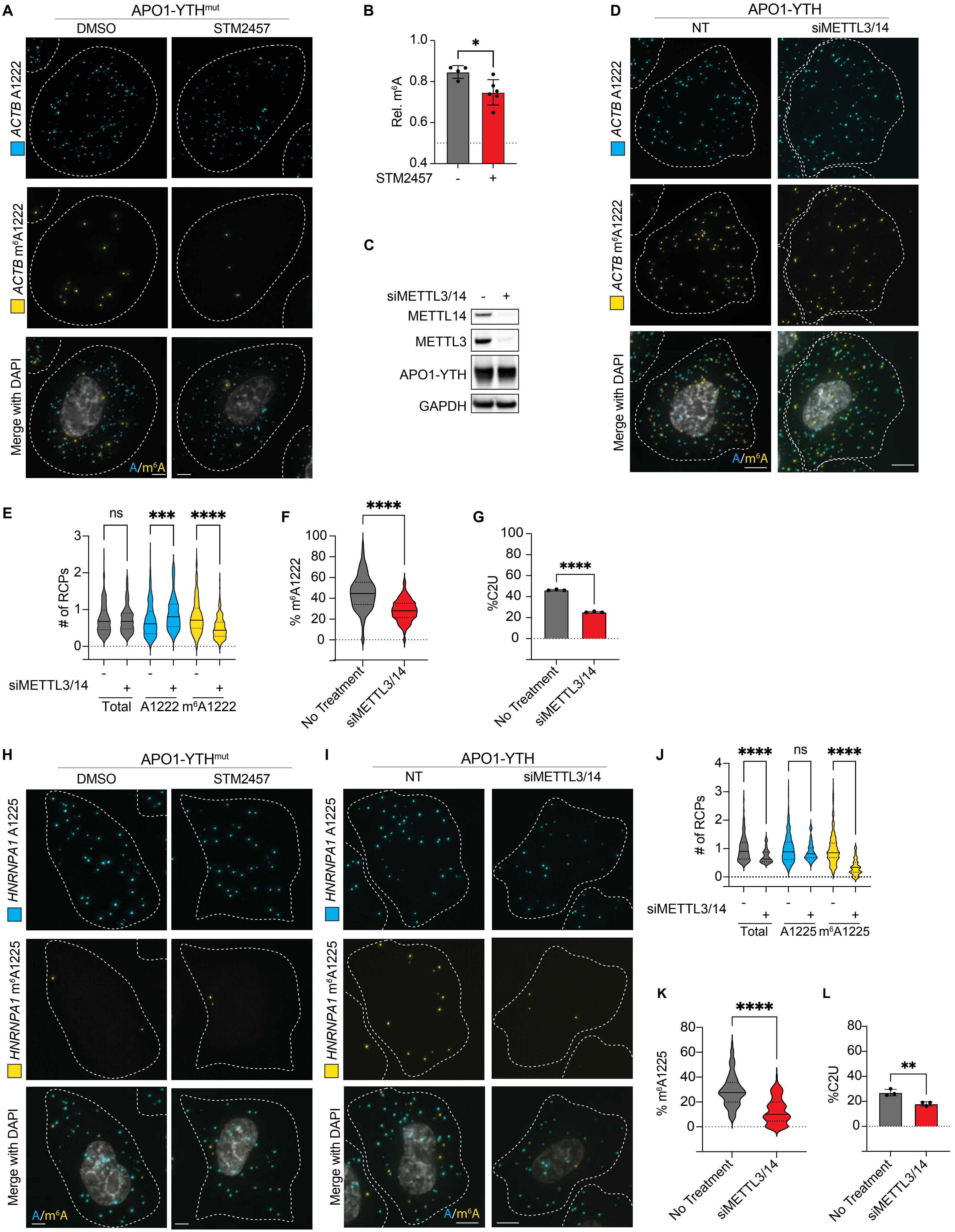

Supplementary Figure 2. DART-FISH detection of m^6A sites is METTL3-dependent.

(**A**) Representative images of DART-FISH targeting *ACTB* m^6A1222 in APO1-YTH^{mut}-expressing HeLa cells treated with DMSO or STM2457. Dotted lines represent cell outlines. Scale bar = 5µm. (**B**) RT-qPCR-based m^6A quantification at the *ACTB* A1222 site in HeLa cells treated with DMSO (-) or STM2457 (+). Dotted line indicates the threshold value (0.5) for the presence of m^6A. Values shown are mean ± s.d.; DMSO: n = 4; STM2457 n = 6. *p<0.05. (**C**) Western blot showing depletion of METTL3 and METTL14 in APO1-YTH-expressing HeLa cells following transfection with siRNAs targeting METTL3/14. (**D**) Representative images of DART-FISH targeting *ACTB* m^6A1222 in APO1-YTH-expressing HeLa cells following no treatment or knockdown of METTL3/14. Dotted lines represent cell outlines. Scale bar = 10µm. (**E**) Quantification of the methylated, unmethylated, and total number of *ACTB* A1222 RCPs in individual HeLa cells transfected with siRNAs targeting METTL3/14. Values are plotted relative to the population average in non-transfected cells. Solid lines represent median with quartiles shown as dotted lines. ***p<0.001, ****p<0.0001. ns = not statistically significant. (**F**) Percentage of *ACTB* transcripts with m^6A1222 in individual HeLa cells as detected by DART-FISH following no treatment or knockdown of METTL3/14. Solid lines represent median with quartiles shown as dotted lines. ****p<0.0001. n = 225 for no treatment and n = 147 for siMETTL3/14 for panels E-F. (**G**) %C2U values adjacent to *ACTB* A1222 as determined by RT-PCR and Sanger sequencing in APO1-YTH-expressing HeLa cells following no treatment or knockdown of METTL3/14. Values shown are mean ± s.d. n = 3 for both conditions. ****p<0.0001. (**H**) Representative images of DART-FISH targeting *HNRNPA1* m^6A1225 in APO1-YTH^{mut}-expressing HeLa cells following treatment with DMSO or STM2457. Dotted

lines represent cell outlines. Scale bar = 5μm. (**I**) Representative images of DART-FISH targeting *HNRNPA1* m^6A1225 in APO1-YTH-expressing HeLa cells following no treatment or knockdown of METT3/14. Dotted lines represent cell outlines. Scale bar =10μm. (**J**) Quantification of the methylated, unmethylated, and total number of *HNRNPA1* A1225 RCPs in individual HeLa cells following no treatment or knockdown of METTL3/14. Values plotted are relative to the population average in untreated cells. Solid lines represent median with quartiles shown as dotted lines. ****p<0.0001, ns = not statistically significant. (**K**) Percentage of *HNRNPA1* transcripts with m^6A1225 in individual HeLa cells as detected by DART-FISH following no treatment or knockdown of METTL3/14. Solid lines represent median with quartiles shown as dotted lines. ****p<0.0001. For panels J-K, no treatment: n = 194; siMETTL3/14: n = 104. (**L**) %C2U adjacent to *HNRNPA1* A1225 in APO1-YTH-expressing HeLa cells as determined by RT-PCR/Sanger sequencing. n = 3. **p<0.01.

Supplementary Figure 3

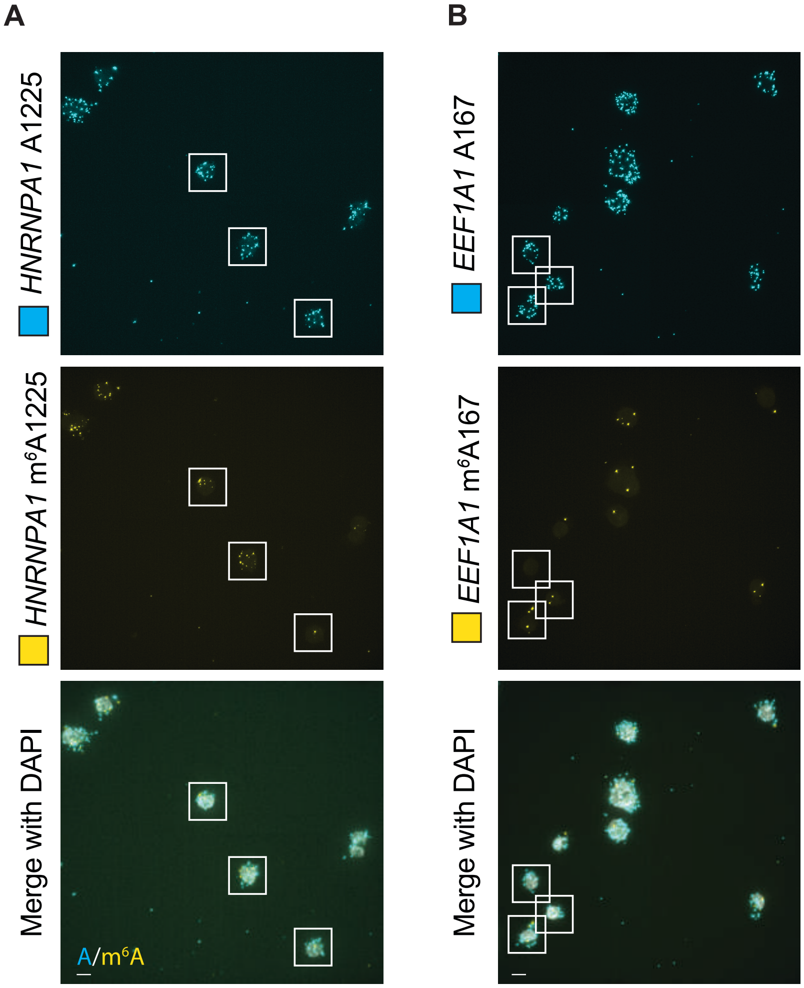

Supplementary Figure 3. DART-FISH measures site-specific m^{6}A methylation in single cells.

(**A**) Whole field of view of DART-FISH images shown in Figure 3A targeting the *HNRNPA1* m^{6}A1225 site in HEK293T cells. Scale bar represents 10μm. Boxed regions indicate individual cells shown in Figure 3A. (**B**) Whole field of view of DART-FISH images shown in Figure 3D targeting *EEF1A1* m^{6}A167 in HEK293T cells. Scale bar represents 10μm. Boxed regions indicate individual cells shown in Figure 3D.

Supplementary Figure 4

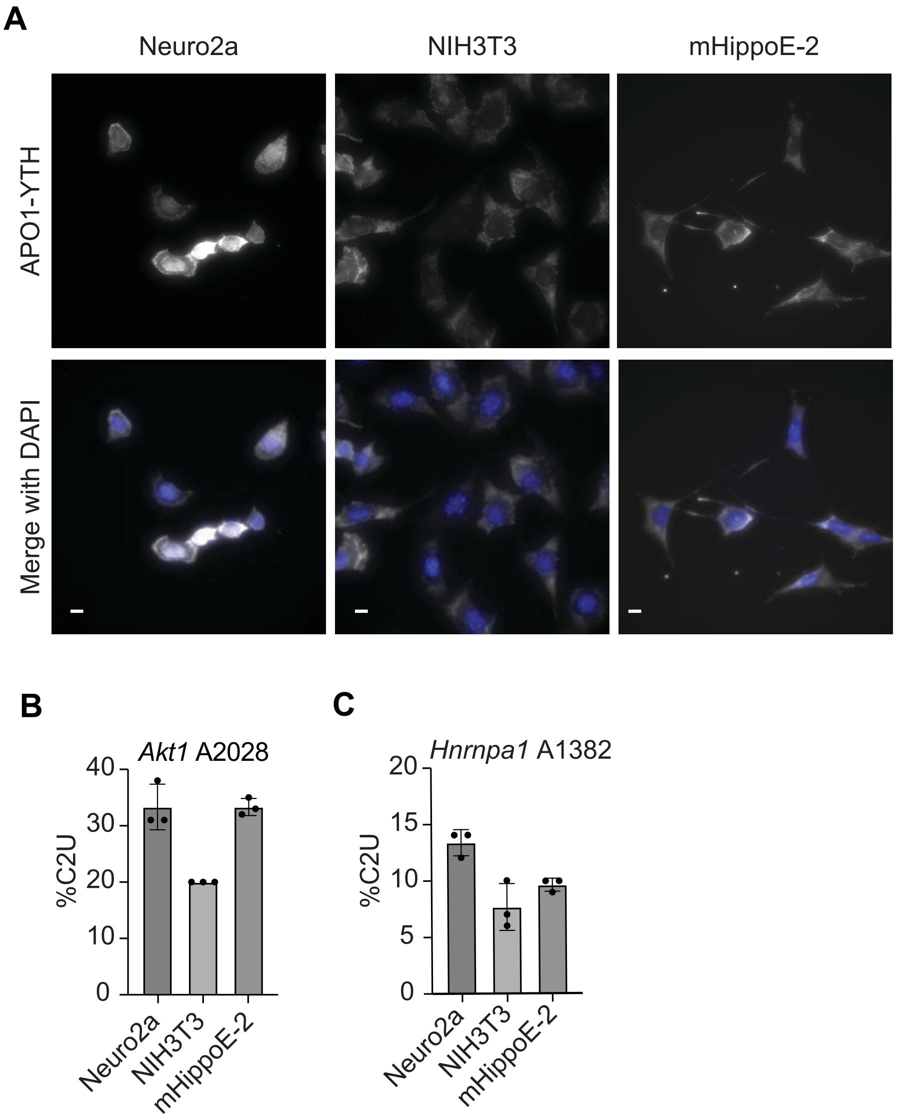

Supplementary Figure 4. Validation of APO1-YTH-expressing mouse cell lines.

(**A**) Representative immunofluorescence images detecting APO1-YTH in Neuro2a, NIH3T3, and mHippoE-2stable cell lines after induction with doxycycline. Scale bar = 10µm. (**B**), %C2U adjacent to *Akt1* A2028 determined by RT-PCR/Sanger sequencing of APO1-YTH-expressing mHippoE-2, NIH3T3, and Neuro2a cells. Values shown are means ±s.d.; n = 3 for all conditions. (**C**), %C2U adjacent to *Hnrnpa1* A1382 determined by RT-PCR/Sanger sequencing of APO1-YTH-expressing Neuro2a, NIH3T3, and mHippoE-2 cells. Values shown are means ±s.d.; n = 3 for all conditions.

Supplementary Figure 5

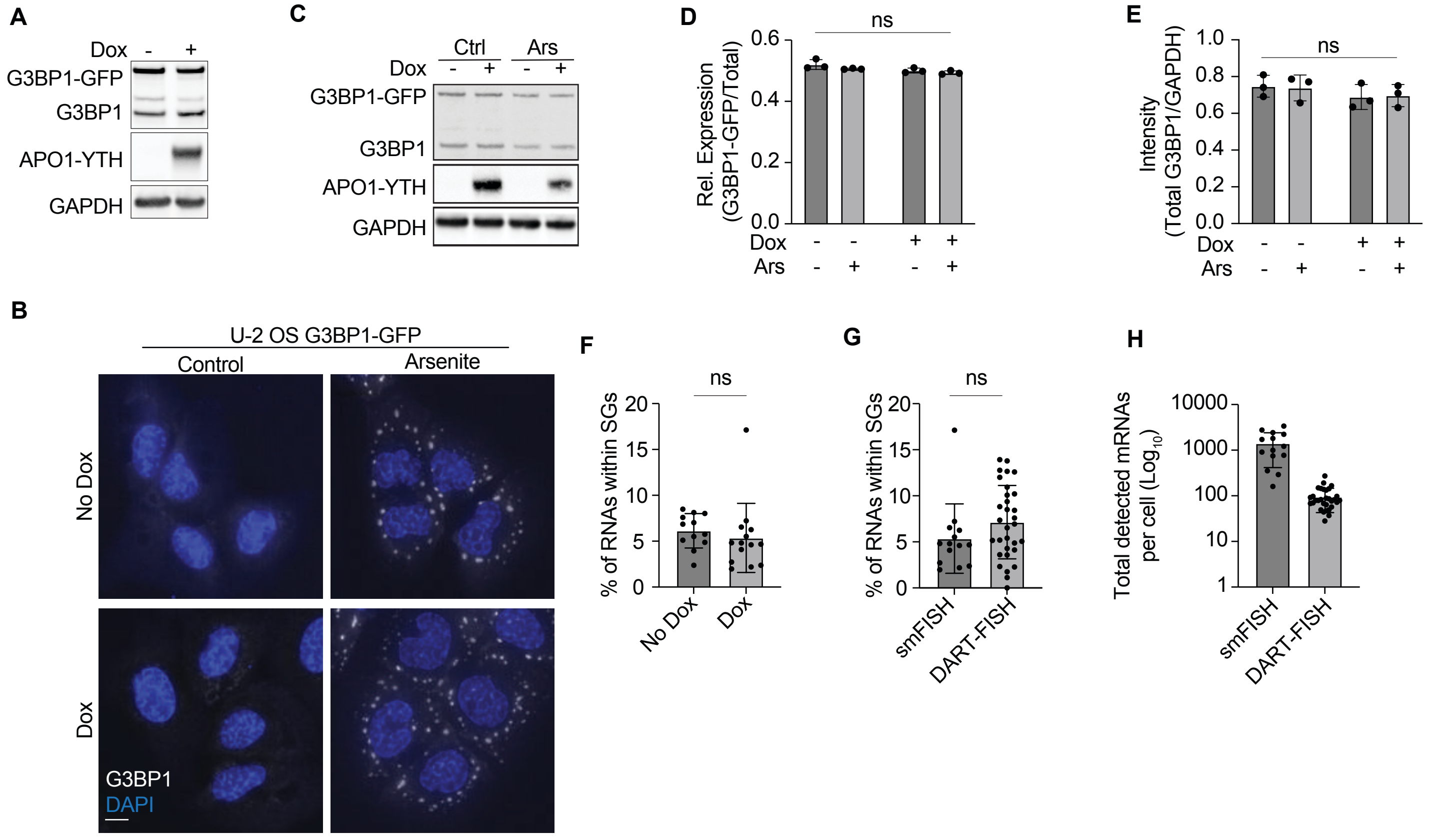

Supplementary Figure 5. Visualization of m^{6}A-modified mRNAs following oxidative stress. (**A**), Western blot from APO1-YTH-expressing U-2OS G3BP1-GFP cells with or without doxycycline induction. (**B**) Representative images of immunofluorescence towards G3BP1 in APO1-YTH U-2 OS G3BP1-GFP cells treated with or without doxycycline (top/bottom) and water or sodium arsenite (left/right). G3BP1 shown in grey, DAPI shown in blue. Scale bar = 10µm. (**C**) Western blot from APO1-YTH-expressing U-2 OS G3BP1-GFP cells treated with or without doxycycline and water or sodium arsenite. (**D**) Quantification of G3BP1-GFP/(G3BP1-GFP + G3BP1) intensity from western blot in panel (C). (**E**) Quantification of total G3BP1 signal (G3BP1-GFP + G3BP1) intensity from panel (C) relative to GAPDH. For panels D-E, n = 3 for all conditions. ns = not statistically significant. (**F**) Percentage of *ACTB* mRNAs colocalized with stress granules determined by smFISH with or without doxycycline induction of APO1-YTH expression. Values plotted are mean ± s.d. Data points represent individual cells. No Dox: n = 12; Dox: n = 14. ns = not statistically significant. (**G**) Percentage of *ACTB* mRNAs colocalized with stress granules determined by smFISH or DART-FISH. Values plotted are mean ± s.d. Data points represent individual cells. smFISH: n = 14; DART-FISH: n = 31. ns = not statistically significant. **H,** Quantification of the total number of *ACTB* mRNA molecules detected per cell by either smFISH or DART-FISH. Values plotted are mean ± s.d. on a Log_{10} scale. Data points represent individual cells. smFISH: n =14; DART-FISH: n = 31.
