# Supplementary material for: In situ visualization of m6A sites in cellular mRNAs
Source: Nucleic Acids Res. 2023 Oct 9;51(20):e101. doi: 10.1093/nar/gkad787 (PMC10639046; doi:10.1093/nar/gkad787)
Supplement: gkad787_Supplemental_Files [file gkad787_supplemental_files.zip › SupplementaryTableLegends.pdf]

Supplementary Table 1. Oligonucleotides sequences.

Listed are the oligonucleotide sequences used in this study given in 5´-3´order. Purpose denotes the experimental procedure the described oligonucleotide was used for.

Supplementary Table 2. Genome coordinates of DART-FISH targeted m^{6}A sites.

Listed are the genome coordinates of the m^{6}A sites targeted by DART-FISH. Sites are given using chromosome (Chr), start, end, and strand notation. End coordinates indicate the position of the m^{6}A-adjacent cytidine residue which undergoes C-to-U deamination and was targeted by DART-FISH. Human sites are provided using the hg38 genome build and mouse sites are provided using the mm10 genome build.
